# Nickel–Zinc Catalysts Favor CO Production via Carbide Formation in CO_2_ Hydrogenation

**DOI:** 10.1021/jacs.6c11100

**Published:** 2026-09-08

**Authors:** Nágila E. C. Maluf, Maitê L. Gothe, Adriano H. Braga, Laís R. Borges, Pedro Vidinha, Alvaro D. Torrez-Baptista, Caetano R. Miranda, János Szanyi, Renato V. Gonçalves, Liane M. Rossi

**Affiliations:** † Departamento de Química Fundamental, Instituto de Química, 28133 Universidade de São Paulo, São Paulo, São Paulo 05508-000, Brazil; ‡ Instituto de Física, DFMT, Universidade de São Paulo, CP 66318, São Paulo, São Paulo 05315-970, Brazil; § Institute for Integrated Catalysis, 6865 Pacific Northwest National Laboratory, Richland, Washington 99352, United States; ∥ Instituto de Física de São Carlos, Universidade de São Paulo, CP 369, São Carlos, São Paulo 13560-970, Brazil

## Abstract

Achieving high CO selectivity in CO_2_ hydrogenation with non-noble metal catalysts remains challenging. Here, we report a simple and cost-effective in situ synthesis of nickel–zinc carbide (Ni_3_ZnC) from Ni species supported on ZnO under reaction conditions. A higher amount of Ni_3_ZnC forms in the calcined NiO/ZnO catalyst compared to the prereduced Ni/ZnO system. Thermodynamic analysis reveals a strong driving force for Ni_3_ZnC formation via NiO/ZnO coreduction (Δ*E* ≈ −455 kJ mol^–1^), which is more exothermic than the metallic Ni pathway due to favorable H_2_O stoichiometry. Density functional theory calculations show that the carbide phase moderates surface reactivity. CO becomes geometrically trapped and isolated from hydrogen due to site blocking by Zn and C atoms. This combination of weakened adsorption and site confinement suppresses deep hydrogenation, leading to high CO selectivity over methanation. This strategy provides a practical route for designing selective reverse water–gas shift catalysts for tandem reaction pathways to liquid fuel synthesis.

## Graphical abstract

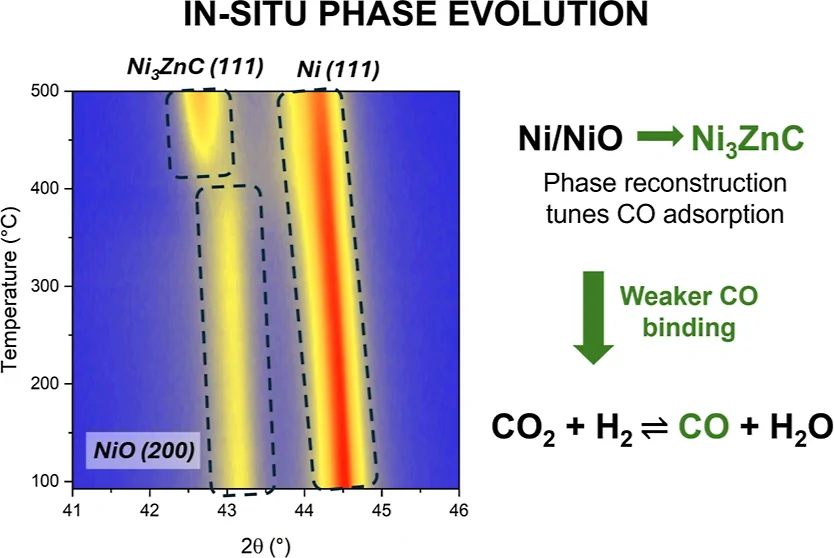

## 1 Introduction

Carbon dioxide contributes nearly 75% of global greenhouse gas emissions, making its capture and utilization essential to combating climate change and ensuring a sustainable future.^1–3^ Among the promising strategies, CO_2_ hydrogenation to value-added chemicals that can be either used as fuels themselves or as feedstock for the chemical industry,^4^ using hydrogen produced from renewable resources^5,6^ and industrial wastes,^7^ is an attractive approach toward a circular carbon economy.^4,8^ Technologies for CO_2_ hydrogenation to C_1_ molecules, mainly CO,^9,10^ CH_4_,^11–13^ and CH_3_OH,^2,14,15^ are well established, while its conversion to C_2+_ products is still challenging. Due to thermodynamic and kinetic limitations, CO_2_ hydrogenation to C_2+_ compounds is envisioned in a two-step process: CO obtained via the reverse water–gas shift (RWGS) reaction (CO_2_ + H_2_ → CO + H_2_O, Δ*H*_298 K_ = 41.1 kJ mol^–1^),^16,17^ that will be further used in Fischer–Tropsch synthesis (FTS) to produce hydrocarbons and oxygenates of medium and long chains.^18–21^ The major challenge of both RWGS and FTS is to avoid the thermodynamically favored methanation reaction.^22,23^ This means that designing catalysts with active sites for RWGS and that simultaneously avoid CH_4_ formation under FTS conditions (high pressures and temperatures in the range of 200–300 °C) is crucial for the development of a two-step process to obtain liquid fuels from CO_2_.

In this context, nickel has been extensively used to catalyze the CO_2_ methanation reaction due to its relatively low price, high abundance, and elevated activity.^24,25^ Although CH_4_ produced from CO_2_ methanation finds interest in the context of renewable energy storage as synthetic natural gas, this product is less valuable than long-chain hydrocarbons and oxygenates. From a catalyst standpoint, promoting strong metal–support interaction in nickel supported on metal-oxides is important to design efficient and stable catalysts, but also to induce a selectivity switch from CH_4_ to CO.^6,26–28^ However, the role of carbon deposition and metal carbide formation on the surface of nickel catalysts during CO_2_ hydrogenation is less explored. Galhardoand Braga et al.^10^ showed that a Ni_3_C-like phase was formed in situ during the CO_2_ hydrogenation reaction over a Ni/SiO_2_ catalyst at atmospheric pressure and temperatures around 600 °C, where the RWGS pathway takes place instead of the methanation pathway, leading to a selectivity switch from CH_4_ to CO. These restructured surfaces were able to suppress CH_4_ formation by weakening the interaction of active sites with adsorbed CO (CO*), which quickly desorbs from the metallic surface instead of undergoing further hydrogenation to CH_4_. Other transition metal carbides (TMCs) have shown a change in selectivity toward the RWGS reaction due to the electronic effect of incorporating carbon atoms into the transition metal interstices. The strong interaction between interstitial carbon and the metal provides an ordered morphology with a modified strained electronic structure that adds noble metal-like behavior to TMCs.^29,30^ The carbon-induced enlargement of the metal–metal bond distances promotes H_2_ dissociation and spontaneous C=O cleavage from CO_2_ adsorbed on the surface, producing the CO* and O* species.^31–35^ Porosoff et al.^36^ demonstrated the activation of CO_2_ in a bent configuration on the surface of Mo_2_C, with further C=O bond cleavage; then, the CO* species desorbs while O* interacts with Mo_2_C to form Mo_2_C–O. Finally, H_2_ completes the catalytic cycle by reducing the Mo_2_C–O and releasing a water molecule, thus avoiding catalyst poisoning.^36,37^

Our group previously explored an intermetallic nickel–zinc carbide (Ni_3_ZnC) material embedded in a N-doped carbon structure, obtained by pyrolyzing the zeolitic imidazolate framework-8 (ZIF-8) impregnated with Ni^2+^ under an inert atmosphere, with nearly complete selectivity toward the RWGS reaction.^17^ The CO_2_ methanation reaction was completely suppressed under atmospheric pressure in the range of temperature studied (up to 500 °C, CO_2_/H_2_ = 1:4). At high pressure in the range of 20 to 100 bar, the selectivity to CO remained above 90%, decreasing slightly with increasing pressure (400 °C, CO_2_/H_2_ = 1:1). The incorporation of carbon atoms via a Ni_3_Zn intermediate to produce Ni_3_ZnC_0.7_ could be obtained under 1% C_2_H_2_/He atmosphere at 200 °C for 1 h^38^ or in situ during CO_2_ hydrogenation reaction.^39^ An analogous intermetallic carbide, InNi_3_C_0.5_, could be obtained by carburizing ZrO_2_-supported In_2_O_3_ and NiO in a stream of a mixture of H_2_ and CO_2_ at 600 °C for 3 h under atmospheric pressure.^40^ Herein, the formation of the Ni_3_ZnC material is described from a simpler route, in which the carbon is added to the material during the CO_2_ hydrogenation reaction. We tested a zinc oxide-supported Ni catalyst for the CO_2_ hydrogenation reaction at ambient and high-pressure conditions, which could represent a cheaper and straightforward strategy to access the Ni_3_ZnC carbide phase to design a highly selective RWGS catalyst. Herein, we demonstrate through experimental and computational approaches that Ni_3_ZnC active sites formed during CO_2_ hydrogenation are more selective to CO than those of Ni^0^ and Ni_3_C.

## 2 Experimental Section

### 2.1 Chemicals

All solvents and reagents used for catalyst preparation and characterization were of analytical grade and used without further purification. Zinc oxide nanopowder (ZnO, <100 nm) and nickel acetate tetrahydrate (Ni­(OAc)_2_·4H_2_O, 98%) were purchased from Sigma-Aldrich (USA), while ethanol (99.5%) was obtained from Synth (Brazil). High-purity gases (N_2_, CO_2_, H_2_, Ar, O_2_; 5.0 grade) were supplied by Gama Gases (Brazil).

### 2.2 Catalyst Preparation and Characterization

The catalyst composed of 10 wt % Ni supported on zinc oxide was prepared by the classical wetness impregnation method. First, 0.42 g (1.7 mmol) of nickel acetate tetrahydrate was dissolved in ethanol (60 mL) under magnetic stirring for 30 min at 60 °C. Then, 0.90 g (11 mmol) of zinc oxide was added, and the mixture was stirred for 12 h at room temperature. The solvent was evaporated under reduced pressure at 40 °C in a rotary evaporator. The obtained powder was pyrolyzed in a horizontal tubular furnace (WT Industria, Brazil) under continuous flow of pure N_2_ (1 L min^–1^) at a heating rate of 5 °C min^–1^ and held at 800 °C for 2 h. The resulting sample was denoted as Ni/ZnO. Aiming to oxidize all metallic Ni in the sample, the Ni/ZnO catalyst was treated with a continuous flow of synthetic air (0.5 L min^–1^) in a vertical tubular furnace (WT Industria, Brazil) at a heating rate of 10 °C min^–1^ and held at 800 °C for 30 min. The oxidized sample was denoted as NiO/ZnO.

The metal content of the Ni catalysts in different life stages was determined using inductively coupled plasma optical emission spectrometry. The samples were digested by alkaline fusion with lithium tetraborate followed by dissolution with nitric acid, and the final solution was analyzed on a Spectro Arcos instrument. CHN elemental analysis was performed for C content estimation using a PerkinElmer 2400 Series II instrument, by heating up to 1000 °C under synthetic air flow. The detection was performed by a thermal conductivity detector that was previously calibrated.

The reducibility of the Ni catalysts was analyzed using temperature-programmed reduction (TPR) on a CATLAB system from Hiden Analytical (UK). Ca. 50 mg of the samples were placed on a fixed bed quartz tube microreactor (length: 200 mm, width: 5 mm) and were first heated up to 150 °C for 30 min under Ar flow, to clean out moisture and other adsorbed species. Then, the sample was cooled down to room temperature, and a mixture of 5 vol % H_2_/Ar flow was admitted to the reactor (30 mL min^–1^), kept for 30 min to ensure stabilization, and the temperature was ramped up to 900 °C. The gas products were measured by online mass spectrometry (Hiden Analytical QGA MS). The commercial zinc oxide nanopowder was pyrolyzed under the same conditions described for Ni/ZnO and used as a reference material.

X-ray diffraction (XRD) patterns were acquired from the samples Ni/ZnO and NiO/ZnO in different life stages to investigate the structure of the catalysts in these different life stages. Measurements were taken in a Rigaku Ultima IV diffractometer, using Cu Kα radiation at 2θ = 10–100° with a 0.02° step size at 40 kV and 40 mA, always at room temperature in ex situ conditions. LaB_6_ powder was previously used for calibration of the line position of the instrument. Rietveld refinement was performed using FullProf software, and a Modified Thompson-Cox-Hastings function was used to fit the peak profile.

Catalyst morphology of the as-prepared and aged Ni catalysts was investigated by high-resolution transmission electron microscopy using a FEI Tecnai G2 F20 microscope for Ni/ZnO and a JEOL 2100 for NiO/ZnO, both operating at 200 kV. The samples were prepared by dispersing the powder in isopropanol with sonication, followed by drop-casting the dispersion onto a carbon-coated copper grid (TedPella). The particle size distribution of Ni species was estimated by counting around 200 particles with the ImageJ software.

### 2.3 Catalytic Tests

CO_2_ hydrogenation reactions were performed under atmospheric pressure in the same Integrated Microreactor-MS system equipped with a CATLAB-PCS Module and a QGA MS Module (Hiden Analytical, UK) used in the TPR experiments. 20 mg (*W*_cat_) of Ni/ZnO catalyst powder was placed in the microreactor (length: 200 mm, width: 5 mm) and pretreated under H_2_ atmosphere (10 vol % H_2_ in Ar balance with a total flow rate of 100 mL min^–1^) at 300 °C for 1 h. Then, the sample was cooled down to 100 °C under Ar flow (50 mL min^–1^); the 1CO_2_/4H_2_ mixture was admitted to the reactor; the gas composition was 2 mL min^–1^ CO_2_, 8 mL min^–1^ H_2_, and 90 mL min^–1^ Ar, giving a gas hourly space velocity (GHSV) of 30 000 mL g_cat_^–1^ h^–1^ (only the reactants gases included in the calculation). The reaction proceeded up to 500 °C, with temperature steps of 25 min at every 50 °C, aiming to reach steady-state conditions. After cooling down, a second reaction cycle was conducted under the same conditions. To investigate changes in selectivity, the catalyst was submitted to CO_2_ hydrogenation reaction in the same reaction conditions described above, maintaining the 1CO_2_/4H_2_ molar ratio and halving the GHSV to 15 000 mL g_cat_^–1^ h^–1^. The oxidized sample NiO/ZnO was submitted to two reaction cycles of CO_2_ hydrogenation as described for Ni/ZnO, using GHSV of 30 000 mL g_cat_^–1^ h^–1^, without a previous reduction. The quantification of reactor outlet was obtained from response factors of the mass spectrometer signals using the Ar signal (*m*/*z* 40 amu) as an internal standard. CO_2_ equilibrium conversion over CO_2_ hydrogenation to CO and CH_4_ was estimated by minimization of Gibbs free energy, considering feeding of CO_2_, CO, CH_4_, and H_2_ at the same conditions as the experimental ones (1 bar, CO_2_/H_2_ molar ratio of 1:4).

High-pressure flow reactions were performed in a stainless-steel tubular reactor (ID: 3.1 mm). A weight of 275 mg of the Ni/ZnO catalyst powder was placed in the reactor between acid-washed quartz sand below and above to hold it in the heated zone of the tubular furnace (WT Industria, Brazil). The catalyst was treated by a continuous flow of H_2_ at 300 °C for 1 h, and then the reactor was fed with a pressurized gas mixture of CO_2_ and H_2_ using a syringe pump of 260 mL (Teledyne Isco 260D). The reactor pressure was controlled with a spring-loaded piston backpressure regulator (Swagelok) connected to a gas chromatography (GC) analyzer system (Shimadzu Nexis GC 2030), to analyze the reaction products in line. The reactions were conducted using a CO_2_/H_2_ molar ratio of 1:1 at 400 °C, with GHSV of 20 000 mL g_cat_^–1^ h^–1^. A control experiment was performed with the reactor packed with acid-washed quartz sand at 400 °C and 100 bar, in which no CO_2_ conversion was observed. CO_2_ equilibrium conversions were estimated from minimization of Gibbs free energy, considering feeding of CO_2_, CO, CH_4_, and H_2_ at the same conditions as the experimental ones (1–100 bar, CO_2_/H_2_ molar ratio of 1:1).

### 2.4 In Situ and Operando Spectroscopy Characterization

The chemical surface composition of the Ni/ZnO catalyst in different life stages was studied by X-ray photoelectron spectroscopy (XPS). In situ XPS measurements were taken using a Physical Electronics Quantera SXM Scanning X-ray Microprobe with Al Kα source for excitation and a spherical section analyzer for “as prepared”, “reduced” at 300 °C for 1 h in 10 vol % H_2_/He at 100 mL min^–1^, and “after reaction” conditions, i. e., at 500 °C for 1 h under 2 mL min^–1^ CO_2_, 8 mL min^–1^ H_2_, and 90 mL min^–1^ He; then cooled down and pumped to ultrahigh vacuum conditions for XPS measurements. XPS spectra were calibrated considering the binding energy of Zn 2p at 1021.7 eV. Data analysis was performed with the CasaXPS software, employing a Voigt function for peak fitting. Previously to the measurements, a low-energy electron flood gun was used for charge compensation.

Structural changes in the Ni/ZnO catalyst were also followed by XRD under operando conditions. The XRD patterns were acquired at the PAINEIRA beamline of the SIRIUS storage ring (Brazilian Synchrotron Light Laboratory).^41^ The Ni/ZnO catalyst powder was loaded into a quartz capillary of 1 mm internal diameter, between quartz wool plugs. The capillary was mounted to a sample holder that allows gas feeding and attached to the diffractometer. The measurements were performed in Debye–Scherrer geometry, with a λ of 0.4263 Å, using an area arc-shaped detector (PIMEGA 450C), covering a 2θ range of 3°–100°; the temporal resolution was 40 s. The CO_2_ hydrogenation reaction was carried out on the previously reduced catalyst (300 °C, under 3% H_2_/He flow, for 1 h), ramping from room temperature up to 500 °C, with a 5 °C/min heating rate. The heating was done by a hot air blower placed right above the capillary. Diluted CO_2_ and H_2_ were fed to the capillary for the reaction, at a final composition of 0.65% CO_2_: 2.6% H_2_: 96.75% He, keeping the 1 CO_2_/4H_2_ molar ratio.

### 2.5 First-Principles Calculations

The structural and electronic properties of Ni_3_ZnC were obtained by first-principles calculations based on the density functional theory (DFT) as implemented in the Vienna Ab Initio Simulation Package (VASP).^42^ We used the generalized gradient approximation proposed by Perdew–Burke–Ernzenhof for the exchange–correlation functional.^43^ We took into account the dispersion forces using the rVV10 self-consistent VdW exchange functional.^44,45^ The atomic core potentials were described as a pseudopotential using the projector augmented-wave method.^46,47^ The kinetic energy cutoff was set at 550 eV for all the simulations. We used the Monkhorst–Pack^48^ scheme to sample the Brillouin zones using the following mesh grids: 7 × 7 × 7 for the Ni_3_ZnC bulk and 3 × 3 × 1 for all surfaces for geometric and electronic optimizations. We used the conjugate gradient method (CG) to perform geometric optimizations, applying force convergence criteria of 0.005 eV Å^–1^ for bulk and 0.01 for slabs.^49,50^ The slab models for all crystallographic surfaces were constructed, ensuring a minimum thickness of approximately 13 Å to adequately represent the bulk-like behavior of the catalyst. In all cases, the bottom third section of the slab was kept fixed at the optimized bulk coordinates, while the upper layers and the adsorbates were allowed to relax fully. To prevent spurious interactions between periodic images along the *z*-direction, a vacuum region of at least 15 Å was maintained.

The reaction energy (Δ*E*) of Ni_3_ZnC formation was computed considering three possible carbon sources (CO, CO_2,_ and CH_4_), and two possible nickel precursors, Ni and NiO, represented by the following equations for CO (eqs 1 and 2), for CO_2_ (eqs 3 and 4), and for CH_4_ (eqs 4 and 5)­

1 $${3Ni+ZnO+CO+H}_{2}{\to Ni}_{3}{ZnC+2H}_{2}O$$

2 $${3NiO+ZnO+CO+5H}_{2}{\to Ni}_{3}{ZnC+5H}_{2}O$$

3 $${3Ni+ZnO+CO}_{2}{+3H}_{2}{\to Ni}_{3}{ZnC+3H}_{2}O$$

4 $${3NiO+ZnO+CO}_{2}{+6H}_{2}{\to Ni}_{3}{ZnC+6H}_{2}O$$

5 $${3Ni+ZnO+CH}_{4}{\to Ni}_{3}{ZnC+H}_{2}{O+H}_{2}$$

6 $${3NiO+ZnO+CH}_{4}{+2H}_{2}{\to Ni}_{3}{ZnC+4H}_{2}O$$

For this purpose, the total energies of all species (Ni, NiO, ZnO, H_2_, H_2_O, Ni_3_ZnC, CO, CO_2,_ and CH_4_) were obtained via first-principles calculations within the DFT framework. Spin polarization was included for Ni-containing species due to their magnetic nature. We calculated the reaction energies using the following expression (eq 7), normalized per stoichiometric unit

7 $$\Delta E=\Sigma E_{products}-\Sigma E_{reactants}$$

where Σ*E*_products_ and Σ*E*_reactants_ are the sum of the total energies of products and reactants, respectively. An alternative direct carboreduction pathway under a pure CO atmosphere yielding CO_2_ was also evaluated, and its corresponding thermodynamic parameters are provided in the Supporting Information.

To investigate catalyst low index crystallographic surfaces, we calculated the surface energy, defined as the surface excess free energy per unit area of a particular crystal facet, using the following expression (eq 8)­

8 $$E_{surf}=\left(E_{slab}-NE_{bulk}\right)/2A$$

where *E*_slab_ is the surface slab’s total energy, *E*_bulk_ is the bulk energy per stoichiometric unit, *N* is the number of stoichiometric units in the surface supercell, and *A* is the surface area. To understand the interaction between surfaces and the CO molecule, we calculated the adsorption energies according to the following equation (eq 9)­

9 $$E_{ads}=E_{total}-E_{slab}-E_{CO}$$

where *E*_ads_ is the total energy of the adsorbed system, *E*_slab_ is the energy of the pristine surface, and *E*_CO_ is the gas-phase molecule energy. A negative value for *E*_ads_ indicates that the adsorption is favorable. We compute the electron charge density given by eq 10

10 $$\rho \left(x,y,z\right)={\rho \left(x,y,z\right)}_{total}-{\rho \left(x,y,z\right)}_{slab}-{\rho \left(x,y,z\right)}_{CO}$$

where ρ­(*x*,*y*,*z*)_total_ is the electron density of the surface/molecule system, ρ­(*x*,*y*,*z*)_slab_ is the electron density of the surface, and ρ­(*x*,*y*,*z*)_CO_ is the electron density of the isolated molecule.

## 3 Results and Discussion

### 3.1 Catalyst Characterization

The Ni/ZnO catalyst was prepared using the wetness impregnation method followed by inert atmosphere pyrolysis up to 800 °C to obtain 10 wt % Ni loading (10.3 wt % of Ni, determined by ICP OES), whereas NiO/ZnO was obtained by calcination of Ni/ZnO in air at temperatures up to 800 °C (9.8 wt % of Ni, determined by ICP OES). The reducibility of the catalysts was compared with a zinc oxide sample pyrolyzed under the same conditions. The H_2_-TPR profile of “as-prepared” Ni/ZnO and calcined NiO/ZnO is composed of two reduction peaks, named A (between 350 and 550 °C) and B (between 550 and 750 °C), while ZnO presented only peak B (Figure S1). The peak A is attributed to the reduction of Ni^2+^ → Ni^0^ species^51^ and is located at ca. 430 °C for Ni/ZnO and ca. 490 °C for NiO/ZnO, which matches their respective peaks of water formation. The shift of peak A to a higher temperature in the NiO/ZnO indicates the strong metal–support interactions between the nickel oxide and zinc oxide particles.^52^ Due to the composition of this sample, in which nickel is fully oxidized, the corresponding peak is much more intense than that observed for Ni/ZnO. The complete reduction of the Ni^2+^ species was achieved at ca. 480 °C for Ni/ZnO and ca. 540 °C for NiO/ZnO. There is a very low intensity event of H_2_O formation at ca. 340 °C for Ni/ZnO, which can be associated with the reduction of OH* or with the desorption of H_2_O*. Peak B, observed at approximately 680 °C for all three samples, is associated with the reduction of ZnO to metallic Zn (Zn^0^) and coincides with the corresponding water-formation peaks. This reduction plays a fundamental role in the catalytic properties of metals supported on ZnO, since the metal would react with Zn^0^ to form alloy phases. However, as demonstrated by Qi and Hu,^53^ this reduction leads to weight loss of ZnO via volatilization. They found that ZnO completely disappears by the reductive treatment with H_2_ for 5 h at 700 °C and that some metals, such as Ni, can slow the ZnO-support weight loss due to the prior reduction of these metal oxides and the formation metal-Zn intermetallic compounds. Therefore, we limited the heat treatments (H_2_ reduction and catalytic tests) to milder temperatures, not exceeding 500 °C. The Ni/ZnO sample shows a peak at ca. 680 °C of CO formation and an event in the wide temperature range from 250 to 500 °C of CH_4_ formation during H_2_-TPR, with no apparent H_2_ consumption or water formation related, which could be assigned to the reduction of atmospheric CO_2_ adsorbed in oxygen vacancies of the support.^54^

The bulk crystal structure of the as-prepared Ni catalysts was investigated by XRD (Figure S2) and compared to nickel standards (Ni, NiO, Ni_3_ZnC, and NiZn). The peak positions of the ZnO crystalline phase are in good agreement with the literature (ICSD #82029). In addition to the support’s peaks, the XRD pattern of the as-prepared Ni/ZnO catalyst is a combination of metallic Ni phase (Ni^0^, ICSD #260169) and the NiO phase (ICSD #9866), corresponding to 77.3% Ni and 22.7% NiO. The sample calcined in air, NiO/ZnO, has only the NiO crystalline phase. The reduction of the Ni/ZnO sample under H_2_ flow at 300 °C was incomplete, as the NiO crystalline phase remained in the XRD pattern obtained after reduction treatment (ex situ). Although the NiZn (111) peak at 43.2° coincides with the Ni (200) reflection, the absence of the NiZn (200) reflection at 46.7° in the experimental data, along with the presence of the NiO (111) peak at 37°, suggests that the material is primarily composed of NiO rather than NiZn (ICSD#105471).

### 3.2 Catalytic Activity in CO_2_ Hydrogenation Reaction

First, the Ni/ZnO catalyst was tested in the CO_2_ hydrogenation reaction at atmospheric pressure under two different gas hourly space velocities (GHSV), using a CO_2_/H_2_ composition of 1:4 (Figures S3), from 100 to 500 °C. This composition was chosen to maintain the CO_2_/H_2_ ratio favorable for CH_4_ formation. The reaction conducted at GHSV of 15 000 mL g_cat_^–1^ h^–1^ reaches the equilibrium conversion for RWGS at a slightly lower temperature than that conducted at GHSV of 30 000 mL g_cat_^–1^ h^–1^. Therefore, the latter GHSV was chosen for the remaining reactions at atmospheric pressure to prevent CH_4_ formation after reaching the maximum CO_2_ conversion to CO predicted by the estimated thermodynamic equilibrium under similar reaction conditions. The Ni/ZnO catalyst was tested in the CO_2_ hydrogenation after reduction at 300 °C for 1 h. After reaching the maximum temperature (1st run), the catalyst was cooled down and tested in a second reaction under similar conditions (2nd run). The NiO/ZnO catalyst was tested in atmospheric pressure CO_2_ hydrogenation under similar conditions, but without being subjected to a reduction step. The conversion curves obtained as a function of temperature are shown in Figure 1a. CO_2_ conversion started at a higher temperature in the first run for the NiO/ZnO sample, suggesting that the catalysts should be previously reduced to be active at a lower temperature. Interestingly, in the second run, both samples (reduced Ni/ZnO and calcined NiO/ZnO) merged to a common conversion curve starting at a lower temperature, which suggests changes in the catalyst structure under reaction conditions. The yields of CO and CH_4_ as a function of temperature are shown in Figure 1b,c, respectively. All the samples displayed low yields of methane (<5%), although it is the thermodynamic favored product at this temperature range. Both catalysts (Ni/ZnO and NiO/ZnO) exhibited high selectivity toward CO, maintaining a selectivity above 90% over two reaction cycles across the entire temperature range studied, suggesting the formation of a carbide phase.^17^

**Figure 1:**
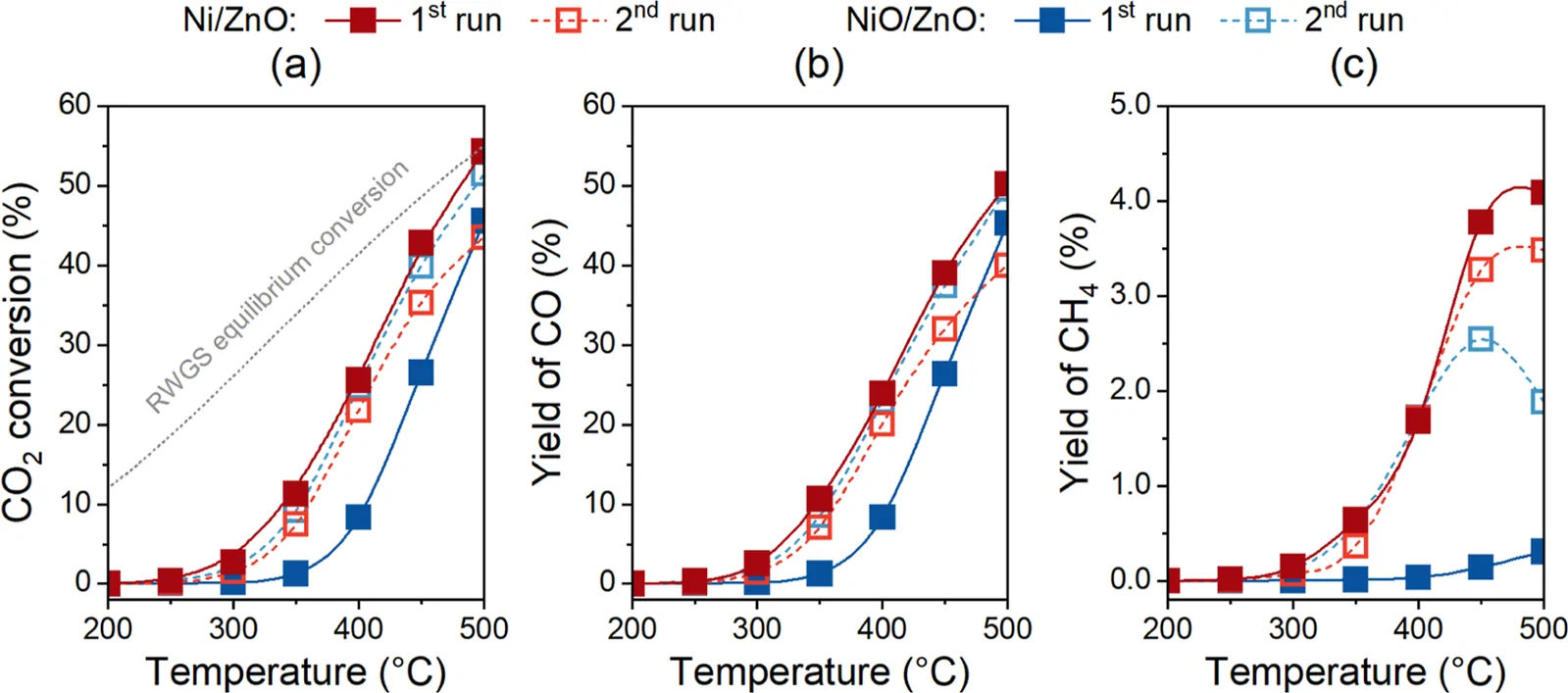
CO_2_ conversion (a), yield of CO (b), and yield of CH_4_ (c) for two successive runs (1st and 2nd runs) of the CO_2_ hydrogenation for the reduced Ni/ZnO catalyst (red) and calcined NiO/ZnO catalyst (blue). (*W*_cat_ = 20 mg, CO_2_/H_2_ = 1:4, total gas flow = 100 mL min^–1^, GHSV = 30 000 mL g_cat_^–1^ h^–1^).

The XRD patterns from 2θ of 41° to 46° of as-prepared, reduced, and spent catalysts are given for comparison in Figure 2a. Both samples, reduced Ni/ZnO and oxidized NiO/ZnO, were converted into the Ni_3_ZnC crystal phase under reaction conditions, as shown by the intense Ni_3_ZnC (111) peak at 2θ of 42.8° (ICSD #77230) in the samples measured after reaction. The NiO (200) diffraction peak present in both samples disappeared, but a less intense Ni (111) diffraction peak remains at a slightly lower 2θ in the Ni/ZnO sample, and it appeared in the NiO/ZnO sample. The presence of metallic Ni, even as a minor phase in both samples, may account for the formation of CH_4_. The presence of the intermetallic Ni–Zn carbide as the major crystal phase is consistent with the observed high selectivity to CO.^17,55^ Although the exact stoichiometry of Ni_3_ZnC_*x*_ may vary with *x* ≤ 1, throughout this work the intermetallic carbide phase is denoted as Ni_3_ZnC, in accordance with the crystallographic standard ICSD #77230. The full XRD patterns of spent catalysts were also compared to standards (Ni, NiO, Ni_3_ZnC, and NiZn) (Figure S4). Consistently, there is no clear evidence of the presence of the NiZn phase, despite the presence of metallic Ni, NiO, Ni_3_ZnC, and ZnO, as confirmed by Rietveld refinement (Figure S6). The results, including *R*-values, cell parameters, concentration of each crystalline phase, peak position, full width at half-maximum (fwhm), interplanar *d*-spacing of the most intense peak of each crystalline phase, and the mean crystallite size (*D*_*hkl*_) estimated by Scherrer, are shown in Tables S1, S2, S3, and S4.

**Figure 2:**
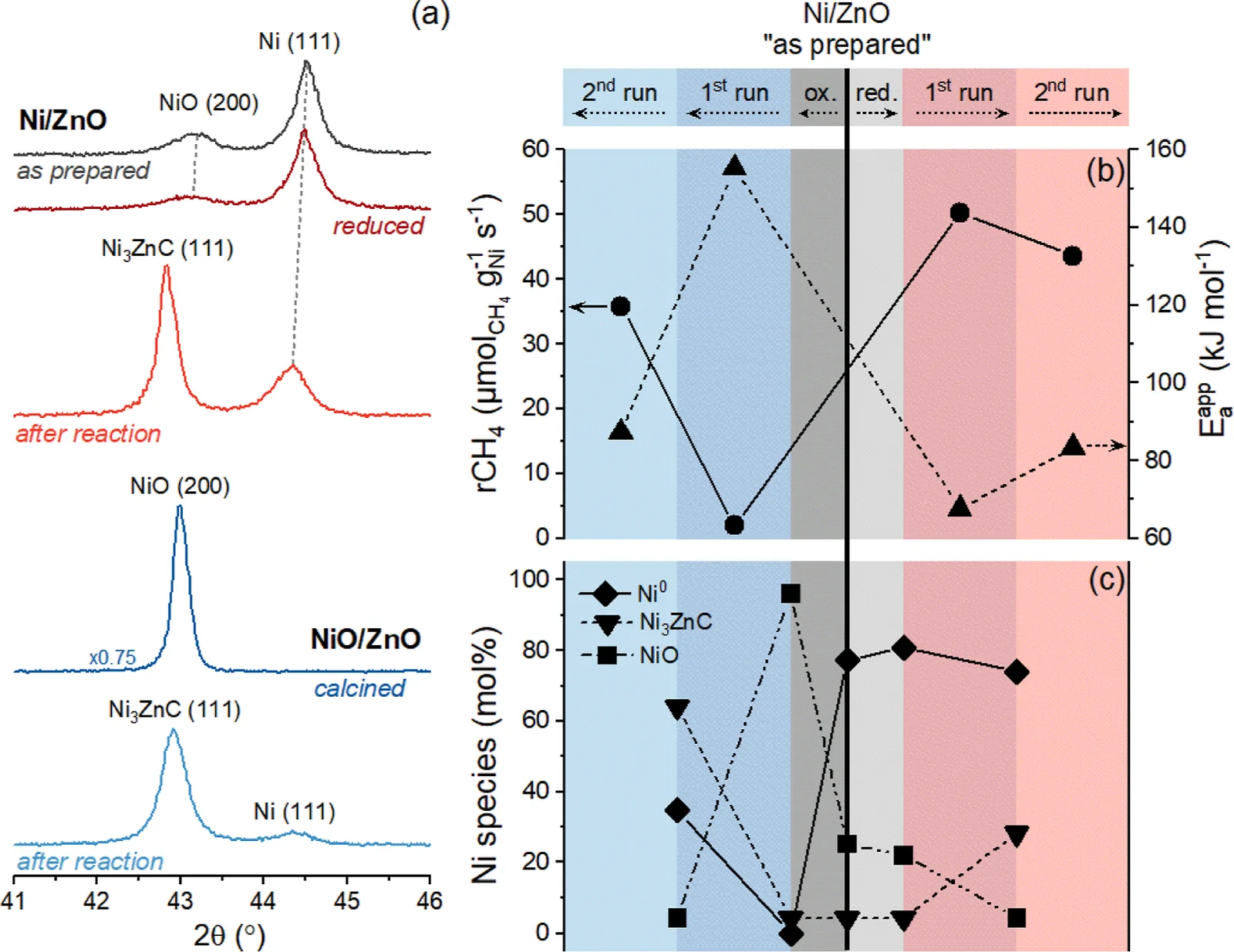
(a) Enlarged XRD patterns of the Ni/ZnO and NiO/ZnO catalysts in different life stages, showing the most intense peak of each Ni-based crystalline phase. (b) Rate of CH_4_ formation at 450 °C versus the apparent activation energy during two runs of the CO_2_ hydrogenation up to 500 °C with a reduced Ni/ZnO catalyst and calcined NiO/ZnO catalyst. (c) Ni species concentration (mol %) estimated by Rietveld refinement of the XRD patterns of Ni catalysts in different life stages. Figure S7 shows a comparison of Ni^0^ (mol %) with Ni^0^ and ZnO crystallite size. (*W*_cat_ = 20 mg, CO_2_/H_2_ = 1:4, total gas flow = 100 mL min^–1^, GHSV = 30 000 mL g_cat_^–1^ h^–1^).

At atmospheric pressure, both catalysts (Ni/ZnO and NiO/ZnO) were highly selective toward CO, but neither catalyst completely suppressed the methane formation. The CH_4_ formation rates (rCH_4_) for the two consecutive reaction runs are shown in Figure 2b. In the reduced catalyst, Ni/ZnO, the rCH_4_ is higher in the first run and only slightly decreases in the second reaction run, while for the oxidized catalyst, NiO/ZnO, it is very low in the first run and increases in the second run. The CH_4_ formation rate directly correlates with the presence of Ni^0^ in the catalyst sample and inversely correlates with the presence of the carbide, Ni_3_ZnC phase (Figure 2c). The presence of the nickel–zinc carbide phase in the catalysts directly correlates with the apparent activation energy (*E*_a_^app^) of the CO_2_ hydrogenation reaction and is associated with decreased nickel activity and lower methanation rates. Arrhenius plots of this reaction are given in Figure S8 and Table S5. The Ni_3_ZnC phase is more efficiently formed when it starts from the oxidized catalyst. The NiO/ZnO catalyst was less active in the first reaction cycle, indicating that the pure oxide phase should be reduced for the reaction to occur. After reduction that occurs in situ by the H_2_ present in the reaction atmosphere, the reaction starts, but at a higher temperature in the first cycle. Moreover, NiO/ZnO was slightly more selective toward CO than Ni/ZnO in the second reaction cycle. NiO/ZnO also improved the level of CO_2_ conversion in the second reaction cycle, while the spent Ni/ZnO catalyst showed lower conversions.

We also studied the Ni/ZnO sample by XRD under *operando* conditions. The same procedure adopted in the catalytic activity measurements (Figure 1) was also applied in the in situ XRD experiment. The reduced sample was put in contact with the reaction atmosphere (1/4 CO_2_/H_2_ ratio), although the reactive gases were diluted in helium for safety reasons. After that, the reaction cell was heated from 100 to 500 °C linearly with XRD data acquisition. Initially, after reduction, the XRD pattern obtained was similar to the ex situ data shown in Figure S2, with a fraction of NiO still remaining in the catalyst (ca. 20 wt % NiO on a nickel basis). Under reaction conditions, any observed phase transformations take place only at ca. 400 °C, a temperature at which the CO_2_ conversion is ca. 8%. At this temperature, a new peak appears at ca. 42.6° and 49.5° (Figure 3a), which can be attributed to Ni_3_ZnC, at reflections (111) and (200) respectively, similarly to those observed at ex situ XRD (Figure S4 and Table S3). Concomitantly, it can be observed that the NiO reflections decrease in the 400–420 °C range, disappearing completely at higher temperatures. Meanwhile, the metallic Ni peaks suffer small changes that become more pronounced when NiO disappears. These observations suggest that the Ni_3_ZnC phase is produced at the expense of NiO motifs, more favorably than from metallic Ni, and keeps being formed later from metallic Ni once NiO is not available anymore. Figure 3b shows the main changes at selected life stages of the catalyst.

**Figure 3:**
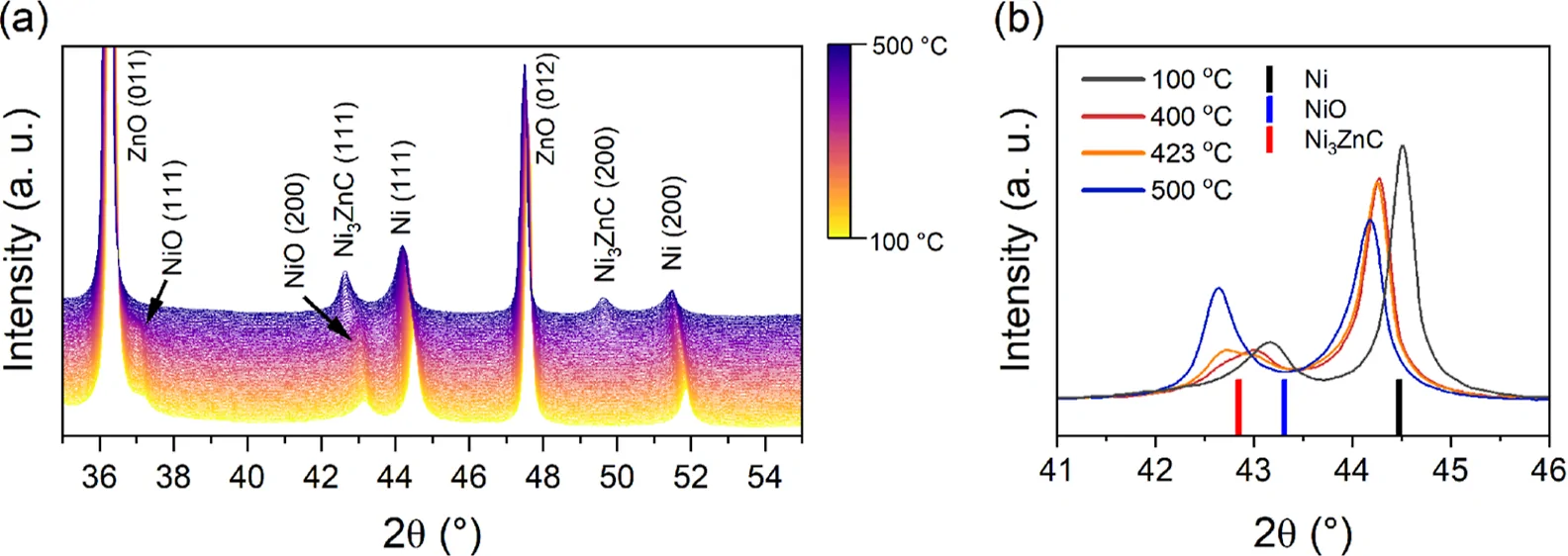
In situ XRD during CO_2_ hydrogenation with a Ni/ZnO catalyst. (a) XRD patterns collected during temperature ramping from 100 to 500 °C and at CO_2_/H_2_ atmosphere (1/4 molar ratio); (b) detailed region showing metallic Ni, NiO and Ni_3_ZnC peaks at the start of the experiment (100 °C), at temperatures where phase transition was observed (400–425 °C range), and at the end of the experiment (500 °C) showing the formation of Ni_3_ZnC and disappearance of NiO. The vertical bars represent the standard peak positions at r.t. for each species; any shift in the experimental data is due to thermal effects.

To verify whether a Ni–Zn alloy could form under reducing conditions, an in situ experiment was performed under H_2_ flow (Figure S5). When Ni/ZnO is heated under H_2_, metallic Ni is the only crystalline phase observed. A tail toward lower diffraction angles can be spotted on Ni/ZnO after heating both under CO_2_ + H_2_ and pure H_2_, although it is more noticeable in the sample exposed to the CO_2_ hydrogenation mixture. This could indicate the dissolution of Zn into metallic Ni to some extent, slightly expanding the lattice parameters. However, a carbon source such as CO_2_ is required for the formation of the ordered intermetallic carbide Ni_3_ZnC, as opposed to a crystalline Ni–Zn alloy being formed first as an intermediate.

The TEM images of the Ni/ZnO catalyst are displayed in Figure 4. In the spent catalysts (Figure 4c,e), Ni nanoparticles can be observed, exhibiting a core–shell structure in which the shell presents a crystalline phase distinct from the core. On the other hand, the Ni/ZnO as-prepared and reduced catalysts (Figure 4a,b) present nanoparticles with a small overlay typical of surface oxidation by sample exposure to air, in which we could not observe isolated FFT patterns due to low crystallinity. The FFT patterns shown in Figure 4c for the spent Ni/ZnO catalyst are in agreement with a core of metallic Ni with lattice fringes of *d*_111_ = 0.204 nm and a shell of a different phase. The surface of the nanoparticle presented in Figure 4c presents a lattice fringe of 0.211 nm, attributed to Ni_3_ZnC (111), which is larger than the NiO (200) lattice fringe (0.209 nm). These findings agree with the crystalline phases found in operando XRD. Additional TEM images of the catalysts are given in Figure S9 in the Supporting Information, along with their size distributions in Figure S10. The particle sizes measured by TEM are compared with Ni^0^ mol % in Figure S11. Elemental composition of the Ni catalysts in different life stages determined by ICP OES and elemental analysis is given in Table S6.

**Figure 4:**
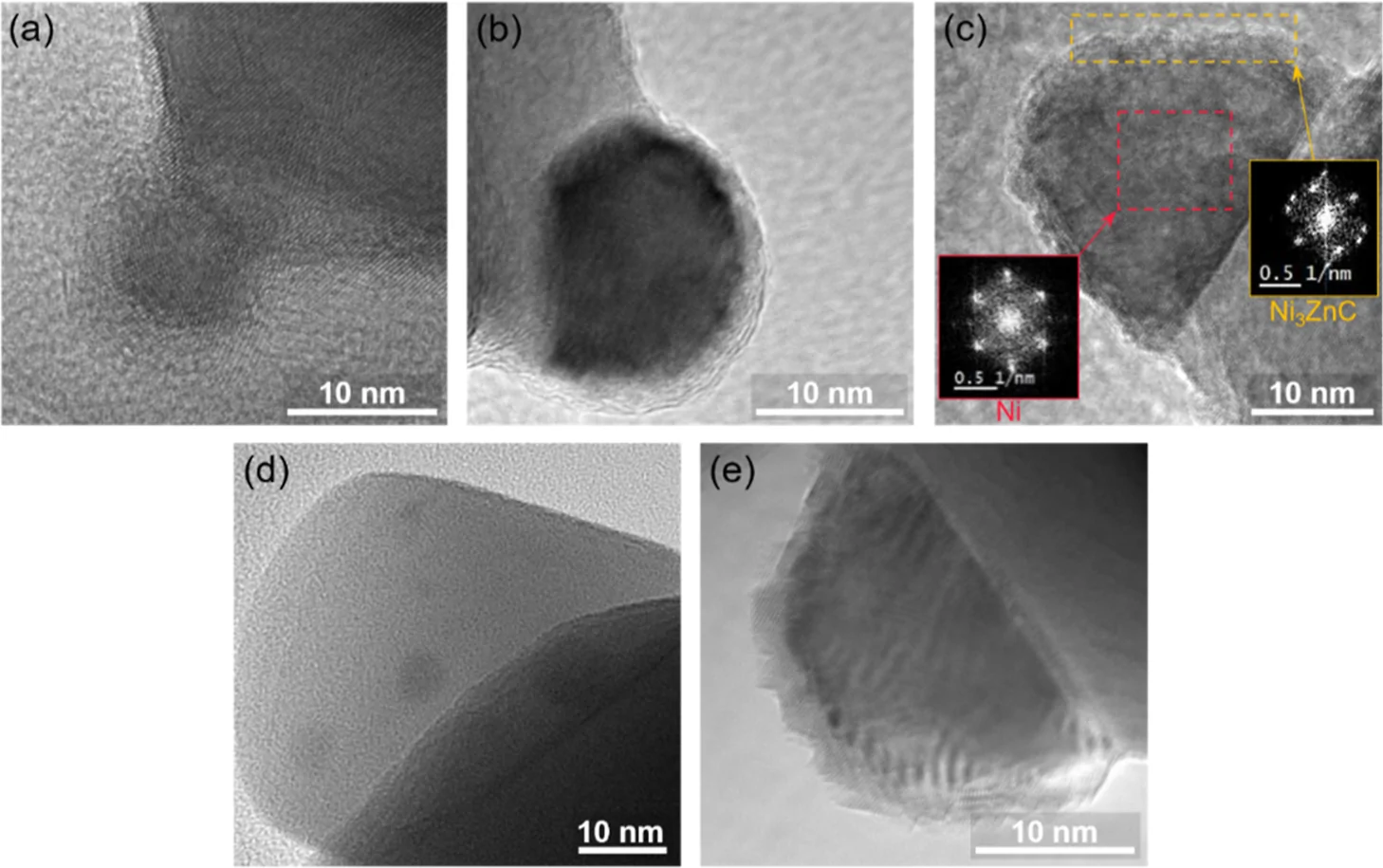
HR-TEM images with FFT patterns for as-prepared (a), reduced (b), and spent (c) Ni/ZnO catalysts, and for calcined (d) and spent (e) NiO/ZnO catalysts.

To further analyze the surface of these materials, Figure 5 shows the C 1s, Ni 2p_3/2,_ and O 1s XPS spectra of the as-prepared, reduced, and spent Ni/ZnO catalyst. The binding energies (BE) and surface composition are presented in Table S7. In the fresh samples, the Ni-supported nanoparticles are composed of metallic nickel and a portion of oxide, as shown by XRD (Figure 2) and TEM (Figure 4). As XPS is highly sensitive to surface, the Ni 2p spectra show two main peaks, at ca. 853.5 and 855.2 eV, corresponding to Ni^2+^ in different chemical environments, the first being attributed to Ni–O and the second one that can be attributed to Ni–OH; in addition, a short component is observed at lower BE of ca. 852.9 eV, that is assigned to metallic Ni.^56,57^ The reduction of Ni/ZnO under H_2_ at 300 °C caused an increase in the metallic Ni component from 8 to 23 at %, at 852.3 eV, yet Ni–O and Ni–OH are still present. On the other hand, after the CO_2_ hydrogenation reaction at 500 °C, the spent catalyst presents only Ni^0^ in the Ni 2p region, while the O 1s region does not show any components related to Ni–O or Ni–OH.

**Figure 5:**
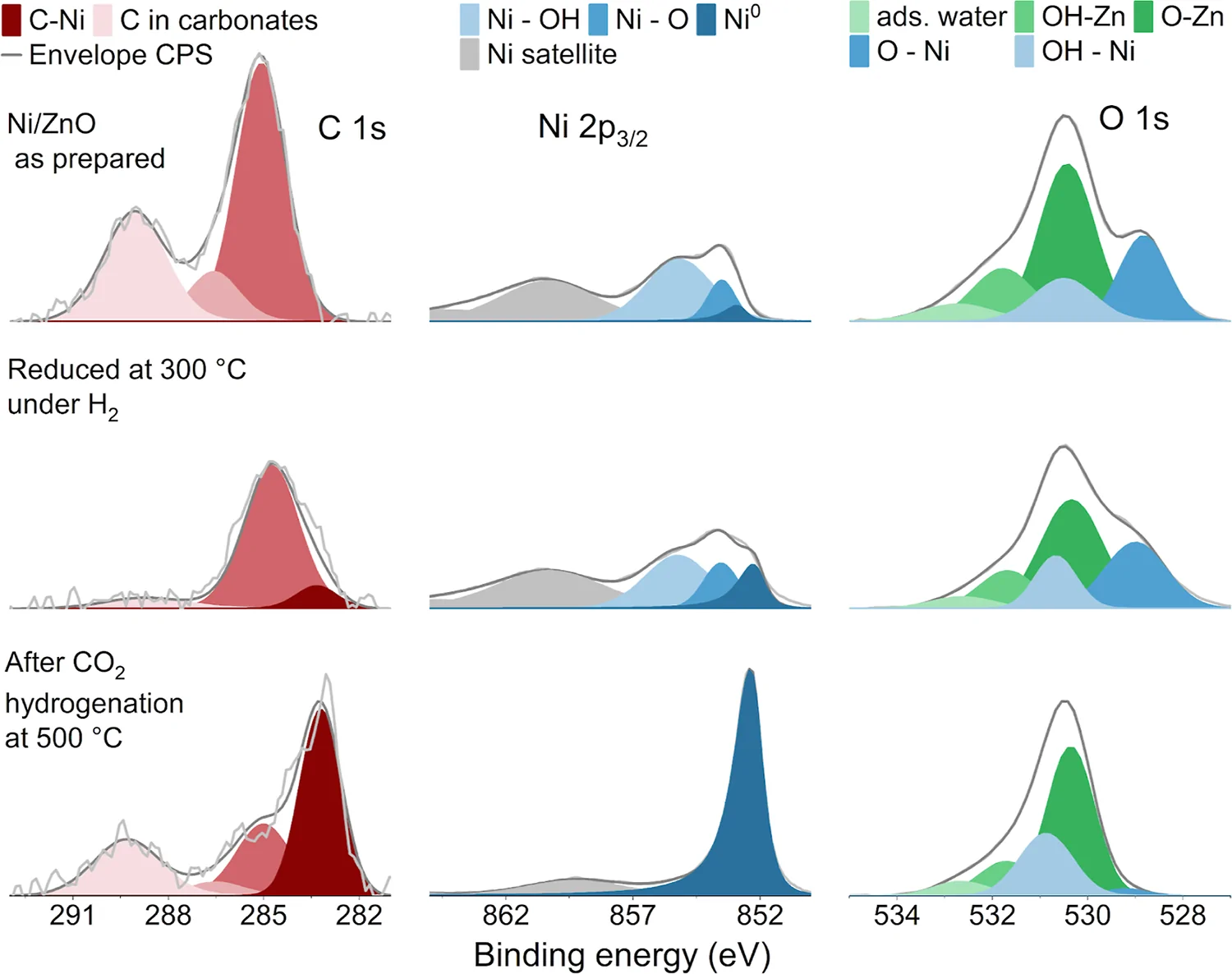
Deconvoluted XPS spectra in the regions of C 1s, Ni 2p, and O 1s of the Ni/ZnO catalyst in different life stages.

The Ni 2p spectra evolution over different stages is similar to what we observed earlier in Ni/SiO_2_ catalysts.^10^ Moreover, in the C 1s spectrum, in addition to adventitious carbon and surface carbonates, a component appears at 283.2 eV of the spent Ni/ZnO catalyst, which can be attributed to a solid solution of carbon interstitially dissolved in metallic Ni with possible formation of a carbide,^10,58^ accounting for 47 at % of the surface carbon on the spent catalyst. This Ni–C species is also observed on the C 1s of the reduced sample prior to reaction, although with only 23 at %. The formation of Ni–C in the reduced catalyst can occur due to the presence of surface carbonates in the as-prepared samples, which can react with the H_2_ reducing atmosphere. Because the Ni–C and metallic Ni BE are close, the Ni 2p_3/2_ peak at 852.3 eV cannot be safely attributed to either of these species. Yet, since there is a significant Ni–C component in the C 1s region, the corresponding species should be visible in the Ni 2p region, and therefore must account for part of the peak at 852.3 eV, if not all of it. These findings reinforce the formation of a nickel carbide phase under CO_2_ hydrogenation reaction conditions.

Considering our previous findings on the high selectivity of Ni_3_ZnC under higher pressure conditions,^17^ the Ni/ZnO catalysts were also tested under increasing pressure (Figure 6). The high-pressure reactions were conducted at a CO_2_/H_2_ ratio of 1:1, in an attempt to inhibit methanation, as a higher H_2_ content was shown to lead to a higher CH_4_ selectivity for the CO_2_ hydrogenation over a similar catalyst. It is quite relevant to obtain information about the behavior of these catalysts under pressure because the conversion of CO_2_ into paraffins or olefins (FTS products) should involve a sequential process, starting with CO_2_ conversion into CO (RWGS). To couple the RWGS and FTS processes in tandem, it would be convenient to operate both at approximately the same pressure, which for FTS is typically >20 bar. However, higher pressures usually favor the CO_2_ methanation reaction instead of the RWGS. Unlike classical Ni/SiO_2_ catalysts, which favor methanation with increasing pressure due to thermodynamic constraints, Ni/ZnO catalysts, containing the Ni_3_ZnC phase, exhibited high selectivity to CO (RWGS) even at pressures up to 100 bar (Figure 6). The CO_2_ conversion slightly increased across the pressure range tested, while the CO selectivity remained higher than 90%, which represents a very promising result. Metallic Ni (Ni/SiO_2_) under similar conditions revealed a selectivity to CH_4_ higher than 90% at 100 bar. No FTS products were observed, and the carbon balance was equal to or greater than 95%.

**Figure 6:**
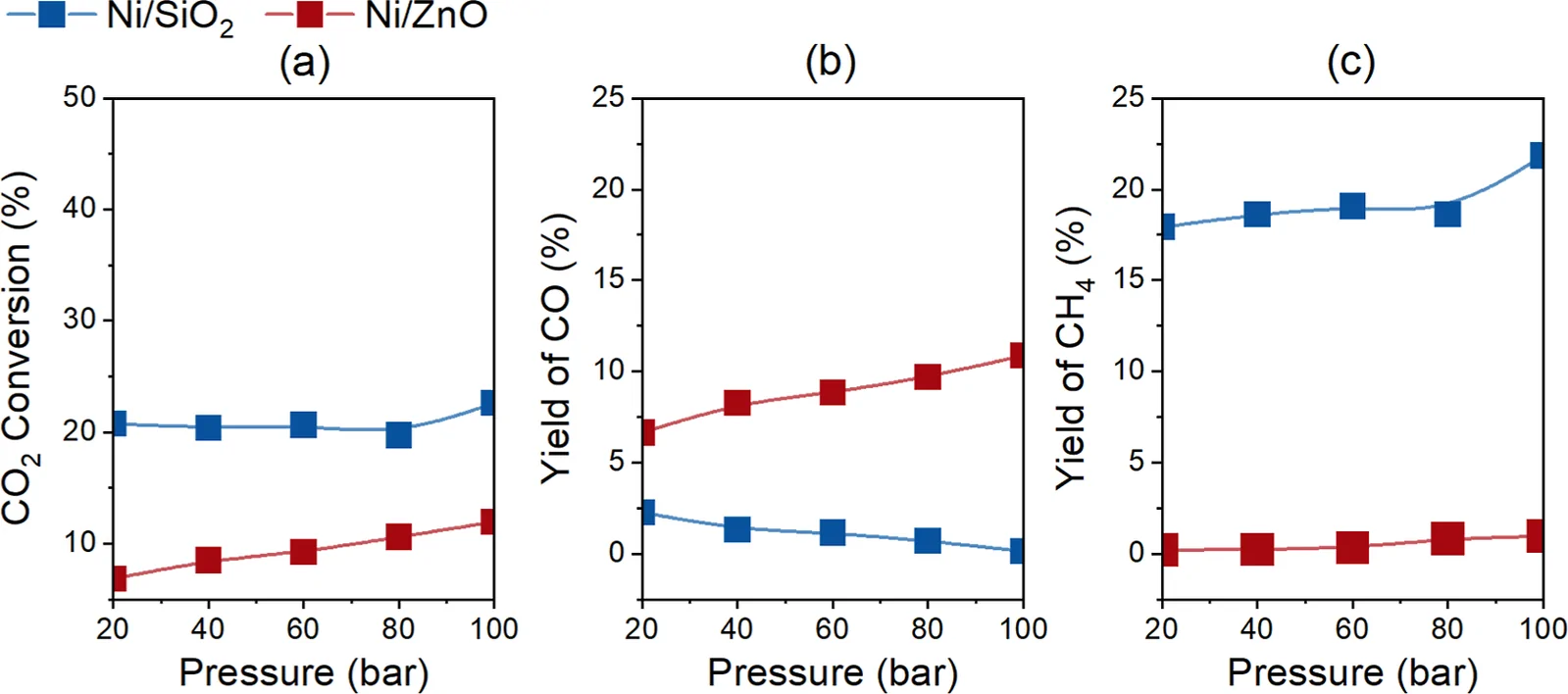
CO_2_ conversion (a), yield of CO (b), and yield of CH_4_ (c) as a function of pressure for the CO_2_ hydrogenation at 400 °C for the reduced Ni/SiO_2_ catalyst (blue) and reduced Ni/ZnO catalyst (red). The samples were pretreated under H_2_ at 300 °C for 1 h. (CO_2_/H_2_ = 1:1, GHSV = 20 000 mL gcat^–1^ h^–1^). Methanol was observed at small amounts (yield less than 0.07%). The carbon balance was between 95 and 99%.

### 3.3 Thermodynamic Analysis of Ni_3_ZnC Formation

The formation of the intermetallic Ni_3_ZnC phase can proceed through distinct solid-state reduction pathways depending on the initial oxidation state of the nickel precursor under a reducing hydrogen (H_2_) atmosphere. To elucidate the experimental synthesis conditions, we compare the thermodynamic feasibility of two competing reactions: (1) the direct reduction of ZnO by H_2_ in the presence of metallic Ni (Metallic route) and (2) the coreduction of both NiO and ZnO precursors (Oxide route). Furthermore, we systematically evaluated three potential carbon sources that may be concomitantly present in the reaction atmosphere: CO, CO_2_, and CH_4_. The calculated reaction energies (Δ*E*) derived from first-principles ground-state optimizations are summarized in Table 1.

**Table 1:** Calculated Reaction Energies (Δ*E*) for Ni_3_ZnC Formation Normalized Per Stoichiometric Unit, Evaluating Different Carbon Sources and Precursor Pathways

C source	precursor route	Δ*E* (kJ/mol)
CO	metallic Ni	–157
	oxide (NiO)	–613
CO_2_	metallic Ni	–86
	oxide (NiO)	–542
CH_4_	metallic Ni	149
	oxide (NiO)	–306

The thermodynamic landscape reveals distinct behaviors depending on both the chemical nature of the carbon carrier and the starting nickel phase. When evaluating CO and CO_2_ as carbon sources, both the metallic and oxide reduction pathways are exergonic (Δ*E* < 0), driven by the structural stability of the resulting intermetallic carbide lattice and the highly favorable formation of H_2_O as a gaseous subproduct. Conversely, when utilizing methane (CH_4_), the metallic route becomes endergonic (Δ*E* = 149 kJ/mol), indicating that the direct carbonization of pure metallic nickel by CH_4_ is thermodynamically hindered under these conditions without additional thermal or chemical driving forces. Regardless of the carbon source employed (CO, CO_2_, or CH_4_), the coreduction pathway starting from NiO precursors consistently exhibits a vastly superior exothermicity compared to its metallic counterpart. Across all three scenarios, the oxide route features a systematic and identical energy downward shift of exactly −455 kJ/mol. From an atomic perspective, this pronounced extra stability arises because the thermodynamic energy released by the reduction of three additional Ni–O bonds and the subsequent formation of three extra H_2_O molecules far outweighs the metabolic cost of cleaving the initial oxide lattices. These results strongly suggest that starting from mixed oxide precursors provides a steep, energetic downhill driving force toward the in situ synthesis of the Ni_3_ZnC catalyst under a reducing atmosphere. This thermodynamic advantage remains robust and invariant to the specific chemical carrier responsible for supplying carbon to the lattice.

### 3.4 Surface Stability and Morphological Hierarchy

To investigate the origin of the CO selectivity in Ni_3_ZnC, we employed first-principles calculations to determine the thermodynamic stability of different crystallographic facets and the strength of CO adsorption. Given the complex unit cell of the Ni_3_ZnC carbide, several surface terminations are possible for each facet. For the (100) surface, we identified two distinct terminations: NiC- and NiZn-ended. Similarly, the (110) surface can be characterized as either Ni- or NiZn-ended. For the (111) facet, two terminations were evaluated, denoted for simplicity as C-ended and NiZn-ended, based on their topmost layer composition. The surface energies (*E*_surf_) calculated using eq 7 are summarized in Table 2. For the (100) facet, the NiC-ended termination was the most stable (1.62 J m^–2^), followed by the NiZn-ended surface (1.67 J m^–2^). The (111) and (110) facets showed intermediate stability, with values ranging from 1.91 to 2.04 J m^–2^.

**Table 2:** Calculated Surface Energies of Ni_3_ZnC for the Four Low-Index (100), (111), (110), and (211)^a^

surface	termination	*E*_surf_ (J m^2^)
100	NiC	1.62
	NiZn	1.67
111	C	1.91
	NiZn	1.99
110	Ni	1.95
	NiZn	2.04
211	NiZn	2.02

a We took into account the possible terminations on each crystallographic surface.

Notably, the stepped (211) surface exhibited a high surface energy of 2.02 J m^–2^, consistent with its undercoordinated and defective nature. Based on these results, the overall stability sequence follows the order: (100) < (111)–(110) < (211). While the (100) facet is thermodynamically favored and likely more prevalent in the polycrystalline catalyst, the inclusion of the higher-energy (211) surface is crucial to model the active step sites typically responsible for the high catalytic turnover and potential selectivity shifts observed experimentally.

### 3.5 CO Adsorption Sites and Energetics and Selectivity Mechanisms

To investigate the chemical affinity of Ni_3_ZnC toward carbon monoxide, we systematically explored the potential adsorption sites on the previously identified stable facets. These sites were classified according to the coordination environment of the CO molecule: top sites, involving linear coordination to a single surface atom; bridge sites, where the molecule is bidentate-bonded to two adjacent catalyst atoms; and hollow sites, characterized by 3-fold coordination within the 3-fold or 4-fold cavities of the lattice. The spatial distribution of these sites on the Ni_3_ZnC terminations is notably more complex than on pristine Ni surfaces due to the presence of Zn and C atoms, which create a chemically heterogeneous landscape. Figure 7 illustrates representative examples of these configurations for each facet combined with their respective electronic charge density variations (Δρ) following eq 10.

**Figure 7:**
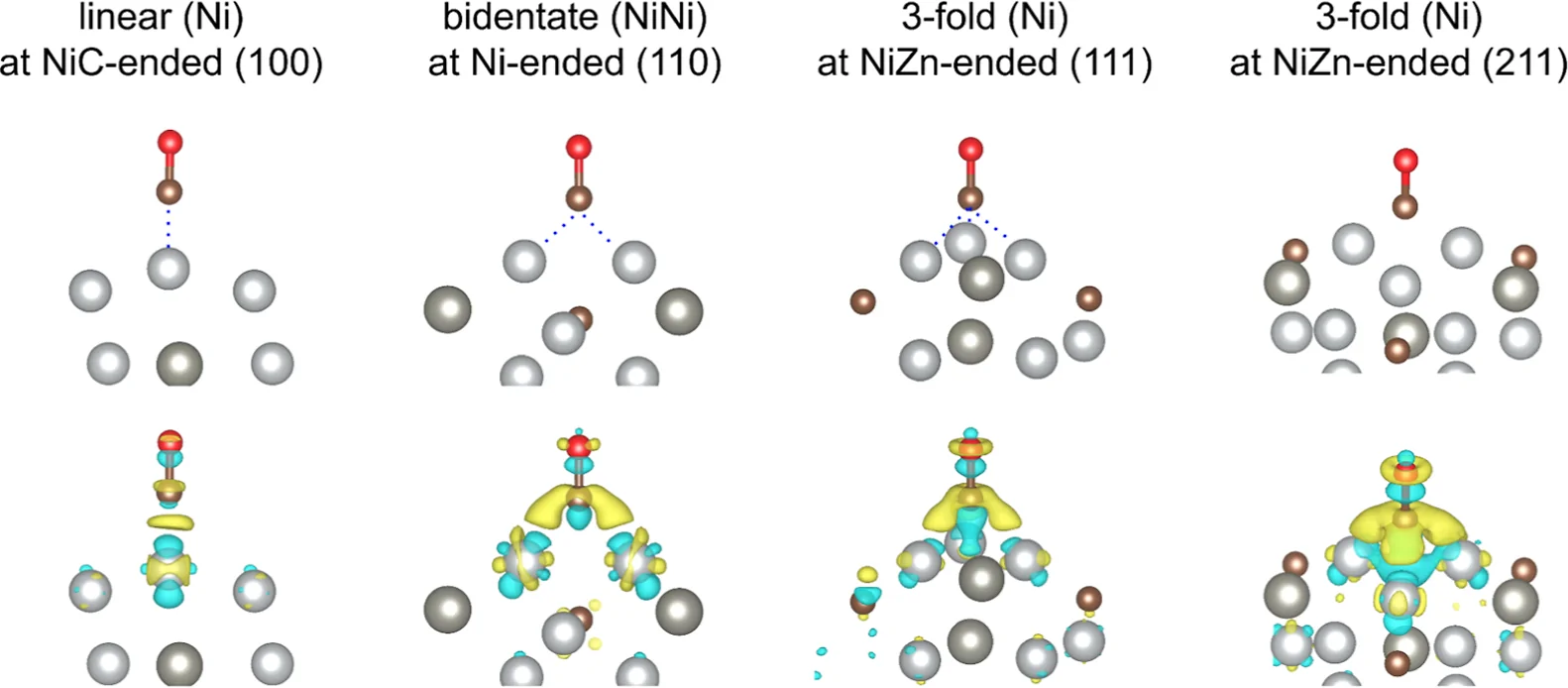
Relaxed CO adsorption structures at Ni_3_ZnC surfaces and charge density differences. The nickel, zinc, carbon, and oxygen atoms are marked as light gray, gray, brown, and red, respectively. The blue and yellow isosurfaces represent charge accumulation and charge depletion regions, respectively. The isosurfaces are shown for 0.009 e/bohr^3^.

The calculated CO adsorption energies (*E*_ads_) for each site are summarized in Figure 8 to identify the preferred binding modes and to compare the reactivity of the carbide phase against reference metallic Ni(111) and Ni(211) surfaces. The global thermodynamic trend indicates that the formation of the Ni_3_ZnC phase significantly moderates the surface reactivity. The majority of investigated sites exhibit *E*_ads_ values that are notably less negative than those of metallic Ni. This generalized weakening of the CO-surface bond is a key descriptor for the high CO selectivity observed experimentally, as it facilitates product desorption and prevents deep hydrogenation steps that lead to unwanted methanation.

**Figure 8:**
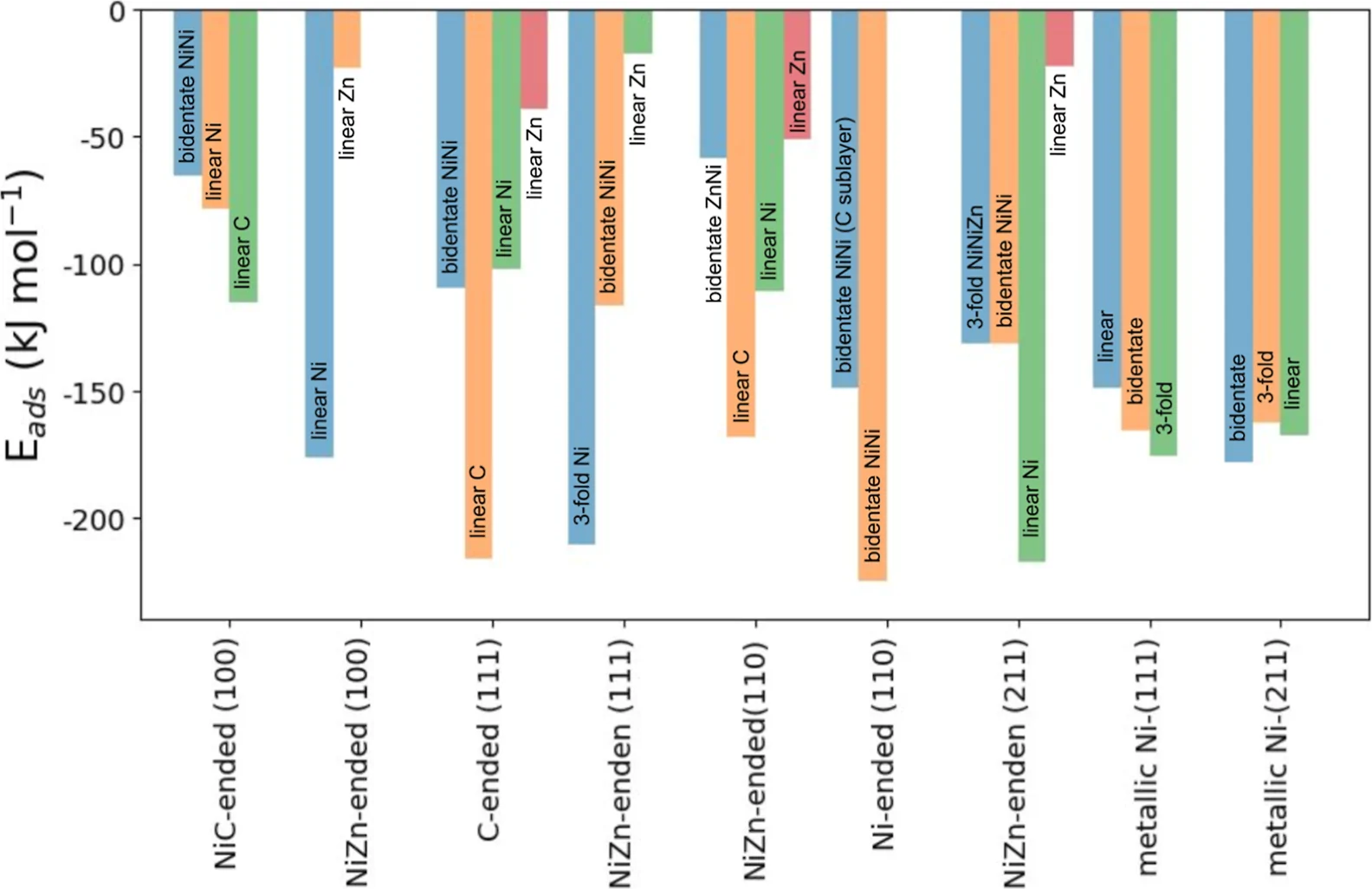
Calculated CO adsorption energies in different sites and topmost layers of three low-index surfaces of Ni_3_ZnC. To compare, we plot the *E*_ads_ on metallic Ni(111) surfaces reported in previous work.^10^

As expected, sites involving direct coordination with zinc (linear Zn) consistently show the weakest chemical interaction with CO (*E*_ads_ > −50 kJ/mol), following trends previously reported for Ni–Zn intermetallics.^59^ For linear adsorption on Ni and C sites, an intermediate behavior is observed. The specific instances where *E*_ads_ values become more competitive with metallic Ni, such as linear Ni at NiZn-ended (100) or linear C at C-ended (111), are explained by multicenter coordination. In these cases, sublayer Ni atoms or surrounding surface atoms participate in a 4-fold or 5-fold coordination (Figures S12 and S13), providing an electronic environment that partially mimetizes the high electron density of pure Ni. Similarly, for bidentate and 3-fold sites, the adsorption is generally weaker than on Ni (111). However, on the Ni-ended (110) surface, the direct exposure of the Ni sublayer leads to a strong bidentate interaction (Figure S14). These findings confirm that while specific geometries can sustain strong bonding, the chemical heterogeneity introduced by Zn and C creates a surface where stable CO adsorption is statistically less favored, thereby promoting product desorption. The inclusion of the (211) surface provides the most compelling evidence for the catalyst’s superior selectivity profile. On metallic Ni (211), the bidentate (bridge) site at the step edge is a deep thermodynamic potential well (*E*_ads_ = −178 kJ/mol) with a C–O bond length of 1.16 Å, a configuration known to activate CO toward dissociation and subsequent hydrogenation. In marked contrast, the chemical heterogeneity of the Ni_3_ZnC (211) step profile drastically alters this trajectory. Geometric optimization reveals that CO undergoes a spontaneous lateral migration along the stepped facet. When initially placed at the edge undercoordinated centers, the molecule tilts away from neighboring Zn atoms to establish a stable, localized 3-fold Ni–Ni–Ni hollow environment (Figure S15). Also, the structural refinement shows that the initial configuration candidate involving a mixed hollow (Ni–Ni–Zn) layout is unstable, relaxing directly into a stable bidentate/bridge Ni–Ni arrangement at the step vicinity. The preferred configuration at the Ni_3_ZnC (211) surface remains the 3-fold Ni hollow site, yielding an *E*_ads_ of −217 kJ/mol. Although this specific energy value is numerically strong, global catalytic selectivity toward CO production over methanation is preserved through two distinct synergistic mechanisms:

i Site dilution (statistical effect): Unlike the pure homogeneous Ni (211) surface, where the entire step edge is highly reactive, the Ni_3_ZnC (211) surface is periodically disrupted by Zn and C atoms. This structural site blocking effectively isolates the remaining active Ni clusters, severely reducing the statistical probability of an adsorbed CO molecule finding adjacent activated hydrogen neighbors required to complete the hydrogenation steps.
ii Electronic modulation and site confinement: The lateral migration into the 3-fold Ni environment is accompanied by a significant elongation of the C–O bond to 1.19 Å (compared to 1.16 Å on the pure metal), indicating a shift in the activation mode. While the molecule is strongly bound to this localized cavity, it is geometrically constrained and electronically trapped. Combined with the severe site isolation of the intermetallic carbide lattice, this confinement effectively prevents further chemical transformations into subproducts, promoting the eventual desorption of clean CO.

Consequently, the synergy between the weakened adsorption on low-index facets and the site confinement on defective surfaces (211) establishes Ni_3_ZnC as a superior catalyst for selective CO production, preventing the deep hydrogenation pathways characteristic of pure nickel.

## 4 Conclusions

A NiO/ZnO catalyst prepared by the classical impregnation method followed by calcination in an inert atmosphere led to a high selectivity to CO in the CO_2_ hydrogenation reaction under both atmospheric and high-pressure conditions. The characterization data of the catalyst before and after reaction showed a structural modification of Ni particles after exposure to reaction conditions at atmospheric pressure, i.e., diluted CO_2_ and H_2_ flow at 500 °C. From Rietveld refinement of XRD data, we deduce that the Ni surface, previously covered with NiO in Ni/ZnO and totally oxidized in NiO/ZnO catalysts, was reduced and enriched with zinc from the support and with carbon from the reactant and/or products of CO_2_ hydrogenation, yielding the Ni–Zn carbide phase (Ni_3_ZnC). A higher proportion of Ni_3_ZnC was observed in the NiO/ZnO spent catalyst. First-principles calculations showed that Ni_3_ZnC formation is more favorable starting from NiO/ZnO than Ni/ZnO, due to a significantly higher exothermicity for the NiO and ZnO coreduction. This simple procedure for the *in situ* formation of the Ni–Zn carbide (Ni_3_ZnC) phase, using such low-cost precursors, represents a novel approach for the preparation of a significantly more selective nickel catalyst than Ni/SiO_2_. Once the Ni_3_ZnC catalyst is stable and maintains a high selectivity to CO with low CH_4_ formation in CO_2_ hydrogenation at atmospheric pressure and at high-pressure conditions, it is a promising candidate for use in RWGS-FT tandem processes for CO_2_-to-liquids applications, even though this low amount of methane might require further processing in industry applications. The analysis of adsorption energies and site-specific interactions on the Ni_3_ZnC surface revealed distinct adsorption behaviors. The weaker binding of CO at Zn-related sites and most bidentate sites suggests reduced surface interactions, facilitating CO desorption. These findings indicate that the Ni_3_ZnC surface offers a balance of sufficient adsorption strength to activate CO but also promotes its desorption, enhancing catalytic selectivity for CO in reaction pathways. This behavior highlights the potential of Ni_3_ZnC as a selective catalyst for processes favoring CO as a product.

## Supplementary Material



## Notes

Additional experimental results and details, including TPR analysis, XRD patterns and Rietveld refinement; TEM images; CO_2_ hydrogenation at different conditions; and in situ XPS data (PDF)
