# Supplementary material for: Nickel–Zinc Catalysts Favor CO Production via Carbide Formation in CO2 Hydrogenation
Source: J Am Chem Soc. 2026 Sep 8;148(37):39923–35. doi: 10.1021/jacs.6c11100 (PMC13614198; doi:10.1021/jacs.6c11100)
Supplement: Supplementary file 1 [file ja6c11100_si_001.pdf]

Supplementary Information

Nickel-Zinc Catalysts Favor CO Production via Carbide Formation in CO_2 Hydrogenation

*Nágila E.C. Maluf[1], Maitê L. Gothe[1], Adriano H. Braga[1], Laís R. Borges[1], Pedro Vidinha[1], Alvaro D. Torrez-Baptista[2], Caetano R. Miranda[2], János Szanyi[3], Renato V. Gonçalves[4], Liane M. Rossi[1] **

[1]Departamento de Química Fundamental, Instituto de Química, Universidade de São Paulo, 05508-000 São Paulo, São Paulo, Brazil

[2]Instituto de Física, DFMT, Universidade de São Paulo, CP 66318, 05315-970, São Paulo, SP, Brazil

[3]Institute for Integrated Catalysis, Pacific Northwest National Laboratory, Richland, Washington 99352, United States

[4]Instituto de Física de São Carlos, Universidade de São Paulo, CP 369, 13560-970 São Carlos, São Paulo, Brazil

*lrossi@iq.usp.br

1. Additional characterization data

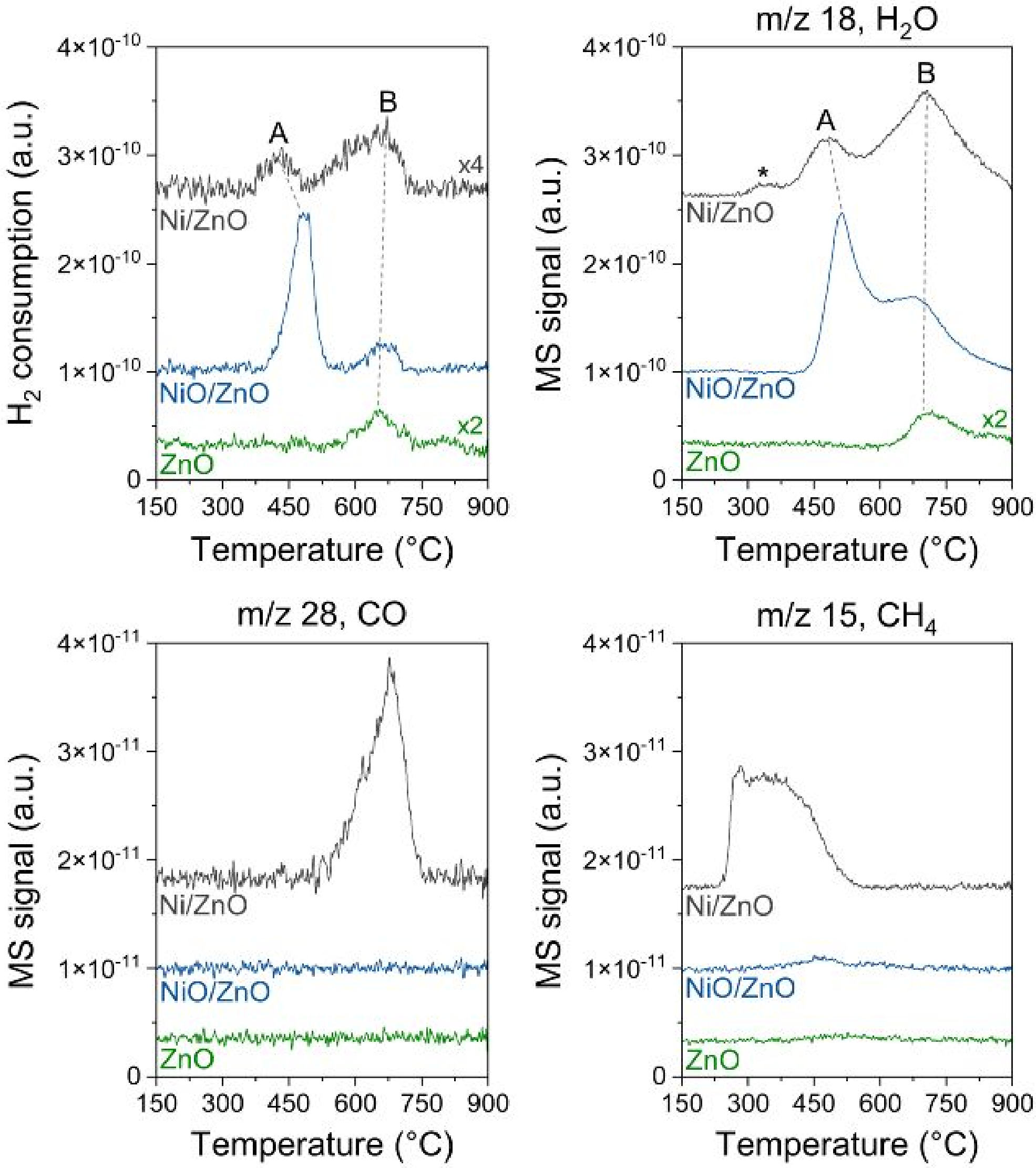

Figure S1: H_2-TPR profiles for "as-prepared" Ni/ZnO (gray line), calcined NiO/ZnO (blue line) and ZnO-Calcined (green line).

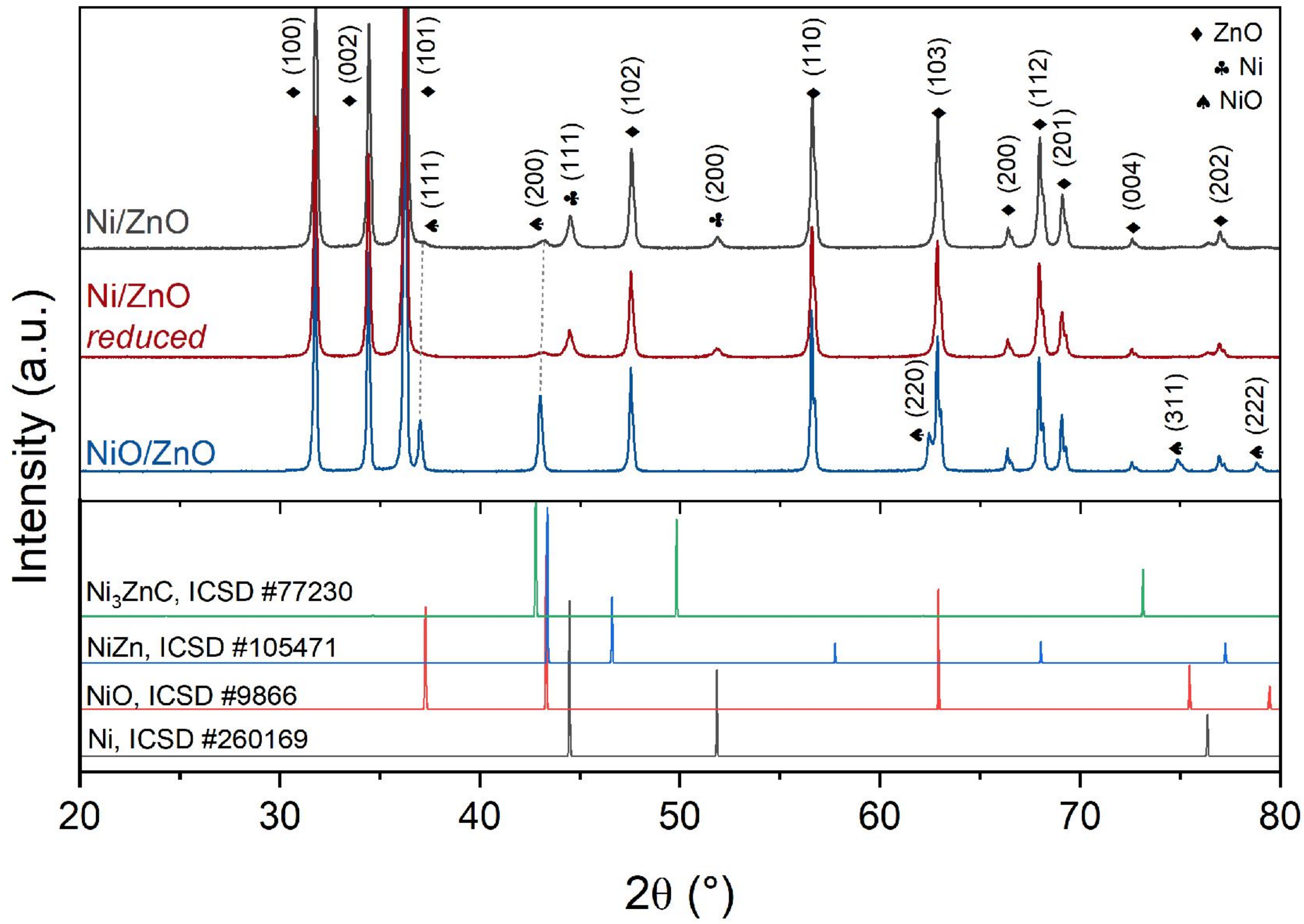

Figure S2: X-ray diffraction patterns of the as-prepared (black line) and reduced (red line) Ni/ZnO catalyst and of the as-prepared NiO/ZnO catalyst (blue line).

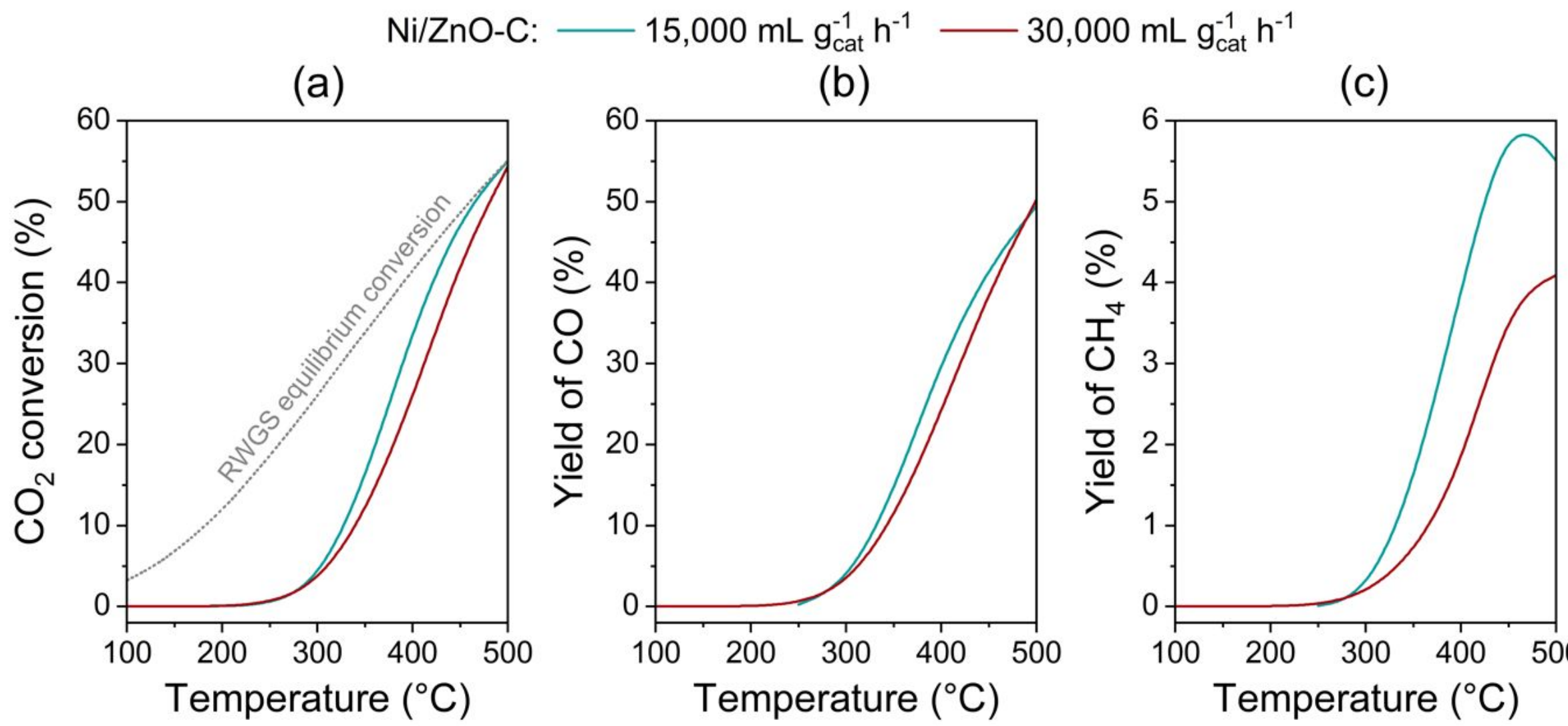

Figure S3: CO_2 conversion (a) and yields of CO (b) and CH_4 (c) as a function of temperature for the CO_2 hydrogenation over Ni/ZnO at different gas hourly space velocity. The sample was

pre-treated under H_2 at 300 ˚C for 1 h. (W_{cat} = 20 mg, CO_2:H_2 = 1:4, total gas flow = 100 mL min^{-1}).

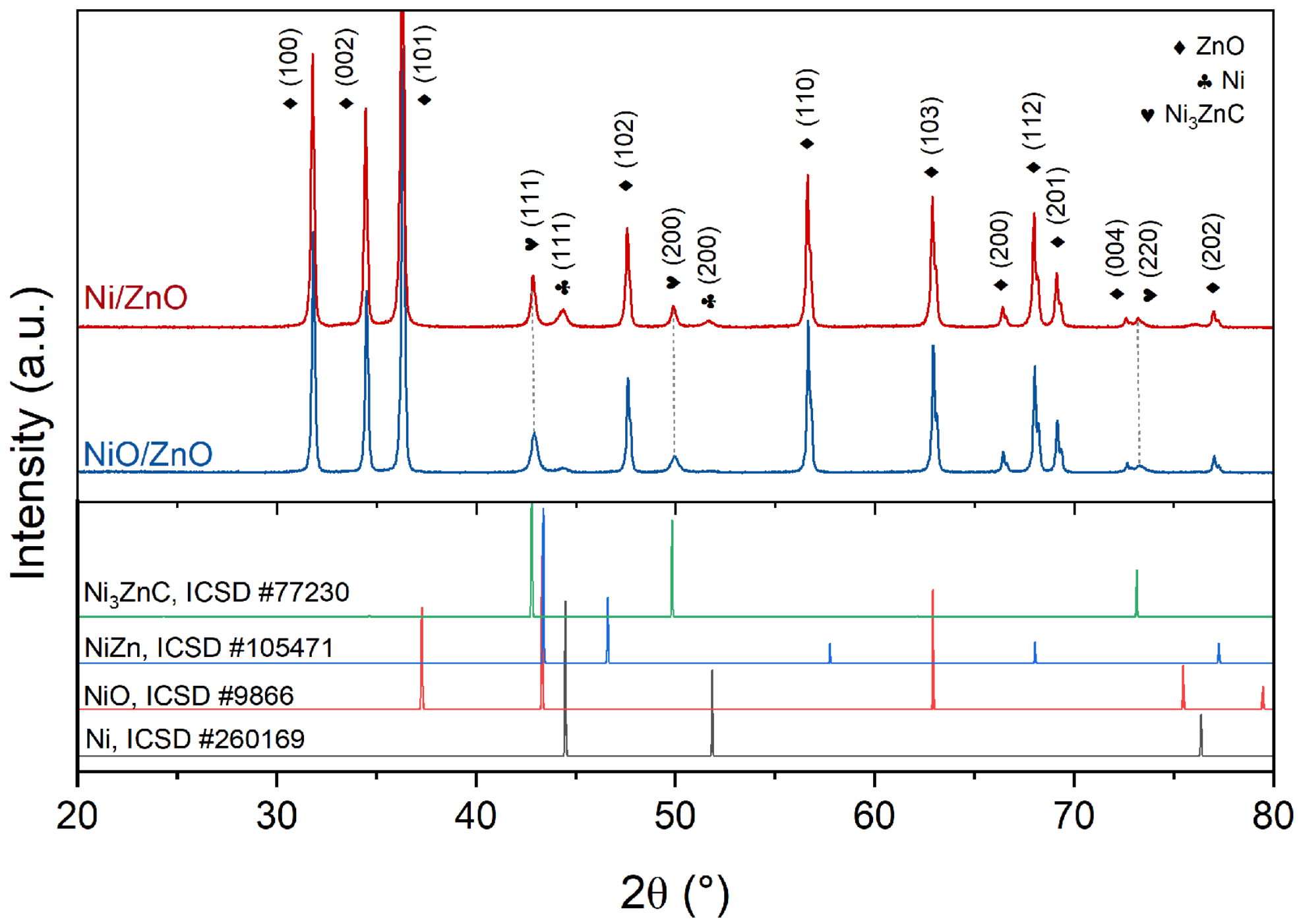

Figure S4: X-ray diffraction patterns of the spent Ni/ZnO (orange line) and NiO/ZnO (blue line) catalysts.

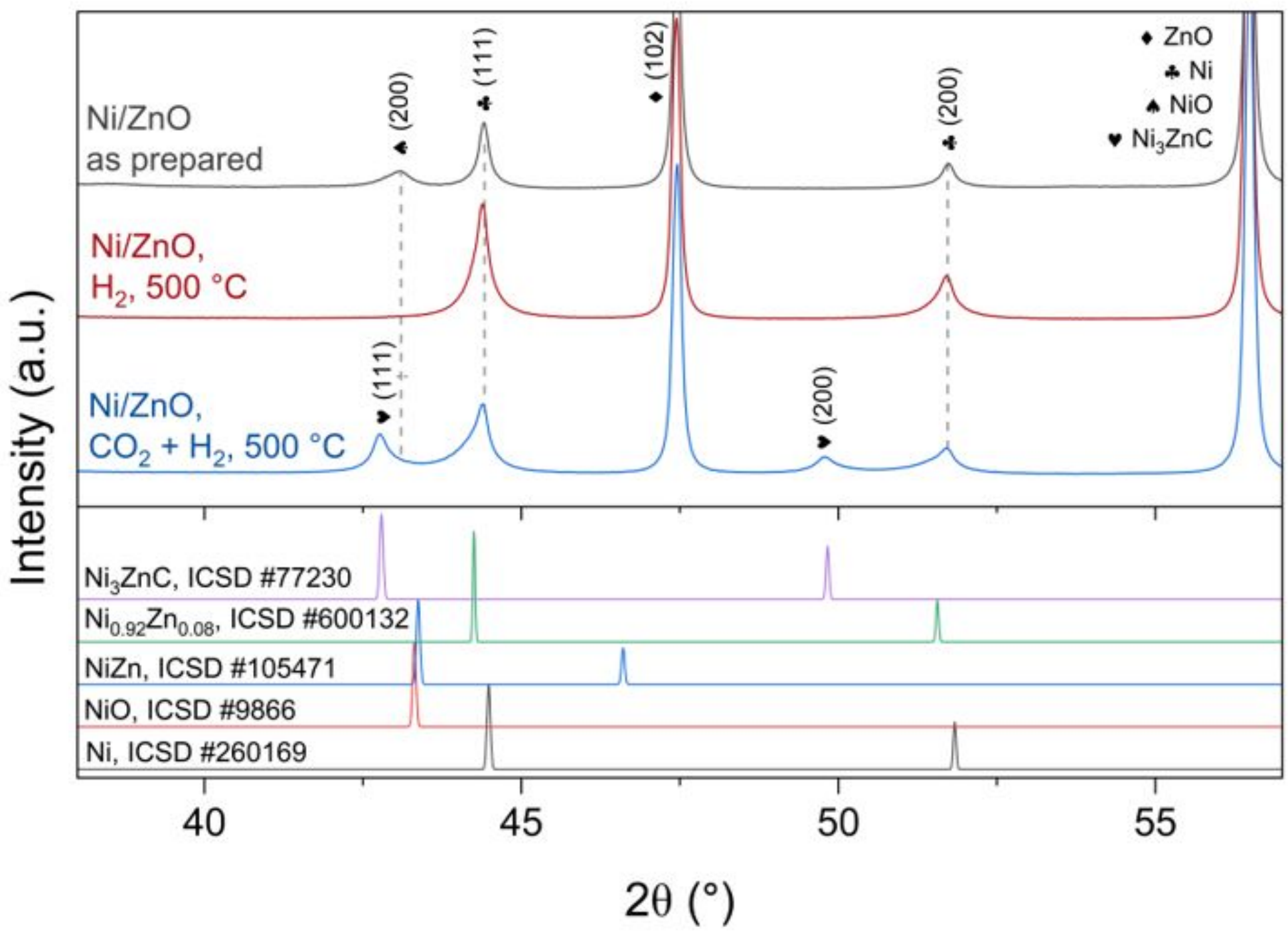

Figure S5: X-ray diffraction patterns of Ni/ZnO measured at room temperature before and after in situ heating up to 500 ˚C under CO_2 + H_2 mixture or only H_2 (diluted in He); λ = 0.48586 Å (synchrotron).

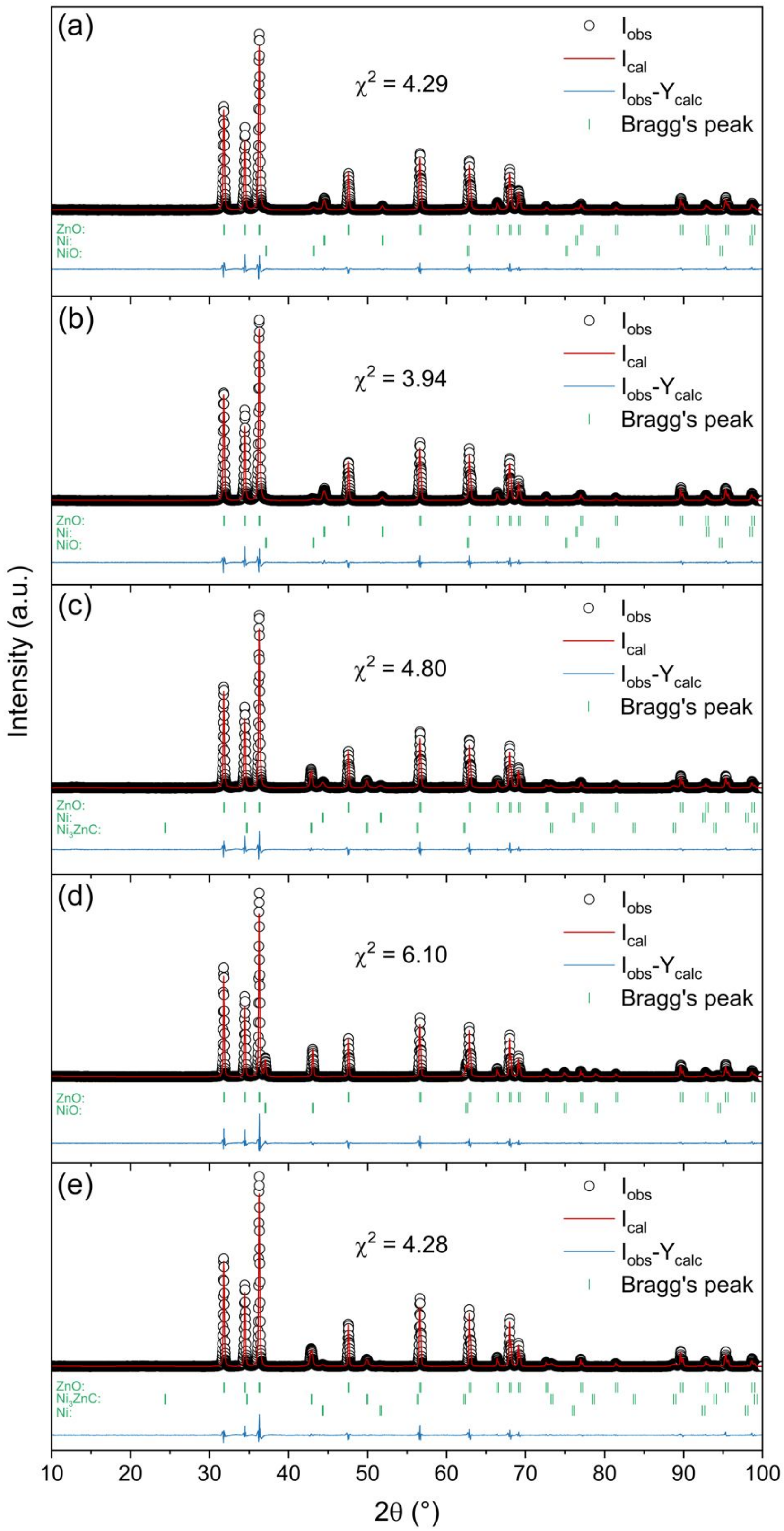

Figure S6: Fitting of the X-ray diffraction patterns calculated by Rietveld refinement for as-prepared (a), reduced (b) and spent (c) Ni/ZnO catalyst and for as-prepared (d) and spent (e) NiO/ZnO catalyst.

Table S1: Rietveld discrepancy values and cell parameters calculated from XRD patterns of the Ni catalysts in different life stages.

Sample	Life stage	Discrepancy values			Cell parameters			
		R_{wp}	R_{exp}	χ^2	*a (Å)*	*c (Å)*	*α (°)*	*γ (°)*
Ni/ZnO	*as prepared*	12.5	6.06	4.29	3.24914[a]	5.20518[a]	90.0[a]	120.0[a]
					3.52455[b]	—	90.0[b]	—
					4.19240[c]	—	90.0[c]	—
	reduced	13.1	6.58	3.94	3.24943[a]	5.20571[a]	90.0[a]	120.0[a]
					3.52488[b]	—	90.0[b]	—
					4.19417[c]	—	90.0[c]	—
	after reaction	13.9	6.37	4.80	3.24907[a]	5.20537[a]	90.0[a]	120.0[a]
					3.53924[b]	—	90.0[b]	—
					3.65432[d]	—	90.0[d]	—
NiO/ZnO	*as prepared*	15.9	6.44	6.10	3.24952[a]	5.20421[a]	90.0[a]	120.0[a]
					4.20132[c]	—	90.0[c]	—
	after reaction	14.0	6.75	4.28	3.24953[a]	5.20468[a]	90.0[a]	120.0[a]
					3.65340[d]	—	90.0[d]	—
					3.54097[b]	—	90.0[b]	—

[a]Refined cell parameters of ZnO crystalline phase (space group: $P6_3mc$, theoretical values: a = b = 3.2490 Å; c = 5.2052 Å; α = β = 90.0°; γ = 120.0°). [b]Refined cell parameters of Ni^0 crystalline phase (space group: $Fm\overline{3}m$, theoretical values: a = b = c = 3.5276 Å; α = β = γ = 90.0°). [c]Refined cell parameters of NiO crystalline phase (space group: $Fm\overline{3}m$, theoretical values: a = b = c = 4.1780 Å; α = β = γ = 90.0°). [d]Refined cell parameters of Ni_3ZnC crystalline phase (space group: $Pm\overline{3}m$, theoretical values: a = b = c = 3.6600 Å; α = β = γ = 90.0°).

Table S2: Phase weight fractions and Ni-based phase molar fractions calculated by Rietveld refinement of the XRD patterns of the Ni catalysts in different life stages.

Sample	Life stage	Weight fraction (wt %)				Ni-based fraction (mol %)		
		ZnO	*Ni*	*NiO*	*Ni_3ZnC*	*Ni*	*NiO*	*Ni_3ZnC*
Ni/ZnO	*as prepared*	91.8	6.0	2.2	—	77.3	22.7	—
	reduced	91.1	6.8	2.1	—	80.9	19.1	—
	after reaction	88.5	4.6	—	6.9	74.0	—	26.0
NiO/ZnO	*as prepared*	83.8	—	16.2	—	—	100.0	—
	after reaction	89.2	1.2	—	9.6	34.8	—	65.2

Table S3: Peak position and full width at half maximum (FWHM) of the most intense peak of each crystalline phase calculated by Rietveld refinement of the XRD patterns of the Ni catalysts in different life stages.

Sample	Life stage	Peak position [2θ (°)]				FWHM [2θ (°)]			
		ZnO[a]	*Ni*[b]	*NiO*[c]	*Ni_3ZnC*[d]	*ZnO*[a]	*Ni*[b]	*NiO*[c]	*Ni_3ZnC*[d]
Ni/ZnO	*as prepared*	36.26	44.49	43.12	—	0.17	0.40	0.42	—
	reduced	36.26	44.48	43.10	—	0.16	0.44	0.88	—
	after reaction	36.26	44.29	—	42.83	0.15	0.55	—	0.25
NiO/ZnO	*as prepared*	36.26	—	43.02	—	0.12	—	0.17	—
	after reaction	36.26	44.27	—	42.84	0.13	0.51	—	0.35

[a]Data from diffraction peak with Miller indices (*hkl*) of 101. [b]Data from diffraction peak with Miller indices (*hkl*) of 111. [c]Data from diffraction peak with Miller indices (*hkl*) of 200. [d]Data from diffraction peak with Miller indices (*hkl*) of 111.

Table S4: Interplanar d-spacing calculated by Rietveld refinement of the XRD patterns and mean crystallite size (D_{hkl}) estimated by Scherrer equation from the most intense peak of each crystalline phase of the Ni catalysts in different life stages.

Sample	Life stage	d-spacing (Å)				D_{hkl} (nm)			
		ZnO[a]	*Ni*[b]	*NiO*[c]	*Ni_3ZnC*[d]	*ZnO*	*Ni*	*NiO*	*Ni_3ZnC*
Ni/ZnO	*as prepared*	2.47	2.03	2.10	—	50.5	22.0	21.5	—
	reduced	2.47	2.03	2.10	—	54.5	20.5	10.1	—
	after reaction	2.47	2.04	—	2.11	57.9	16.4	—	36.0
NiO/ZnO	*as prepared*	2.47	—	2.10	—	74.2	—	52.9	—
	after reaction	2.47	2.04	—	2.11	65.8	17.5	—	25.4

[a]Data from diffraction peak with Miller indices (*hkl*) of 101. [b]Data from diffraction peak with Miller indices (*hkl*) of 111. [c]Data from diffraction peak with Miller indices (*hkl*) of 200. [d]Data from diffraction peak with Miller indices (*hkl*) of 111.

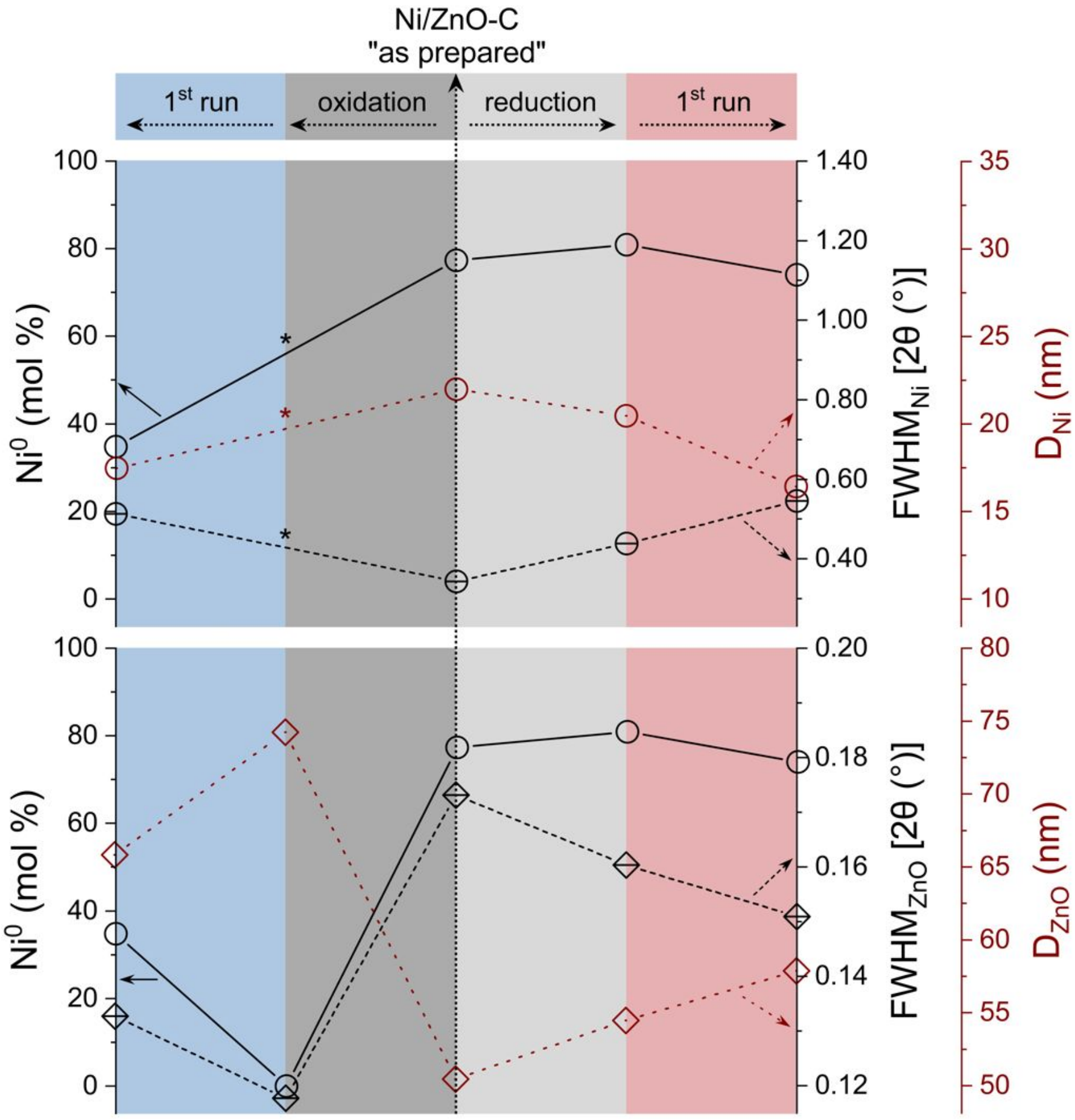

Figure S7: Comparison between the metallic Ni concentration with the full width at half maximum and mean crystallite size from the refined XRD patterns of Ni catalysts in different life stages: data from the most intense diffraction peak of metallic Ni (top) and of ZnO (bottom). *The as-prepared NiO/ZnO has no metallic Ni crystalline phase, so the point was ignored when comparing with the parameters from the peak (111) of Ni^0.

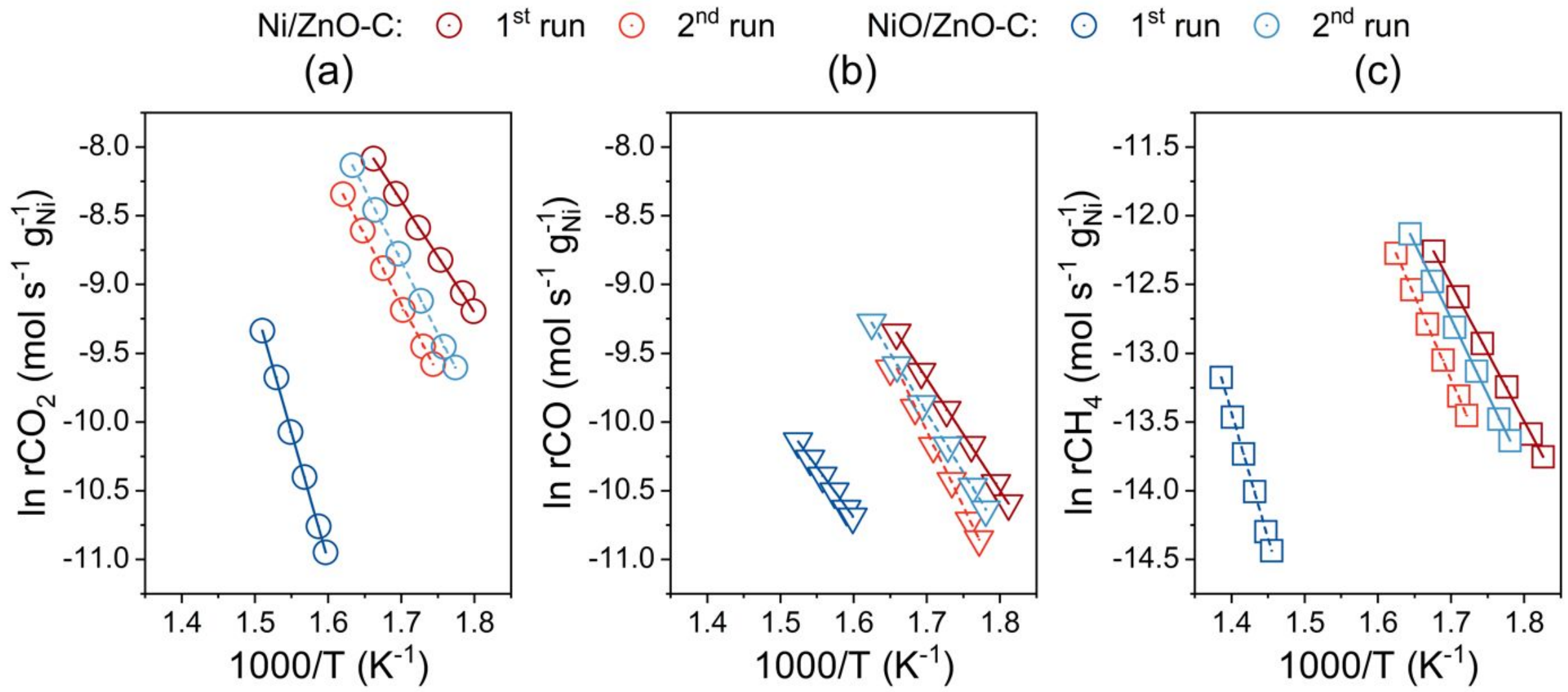

Figure S8: Arrhenius plots of CO_2 consumption rate (a), and of CO (b) and CH_4 (c) formation rates for two runs of the CO_2 hydrogenation over reduced Ni/ZnO and as-prepared NiO/ZnO. (W_{cat} = 20 mg, CO_2:H_2 = 1:4, total gas flow = 100 mL min^{-1}, GHSV = 30,000 mL g_{cat}^{-1} h^{-1}).

Table S5: Apparent activation energy (E_a^{app}) estimated from Arrhenius plots of CO_2 consumption rate for the CO_2 hydrogenation over Ni/ZnO pre-reduced and NiO/ZnO as prepared in the 1st and 2nd reaction runs.

Sample	Run	E_a^{app} (kJ mol^{-1})
Ni/ZnO	*1st*	67.6
	2nd	83.4
NiO/ZnO	*1st*	173.2
	2nd	87.3

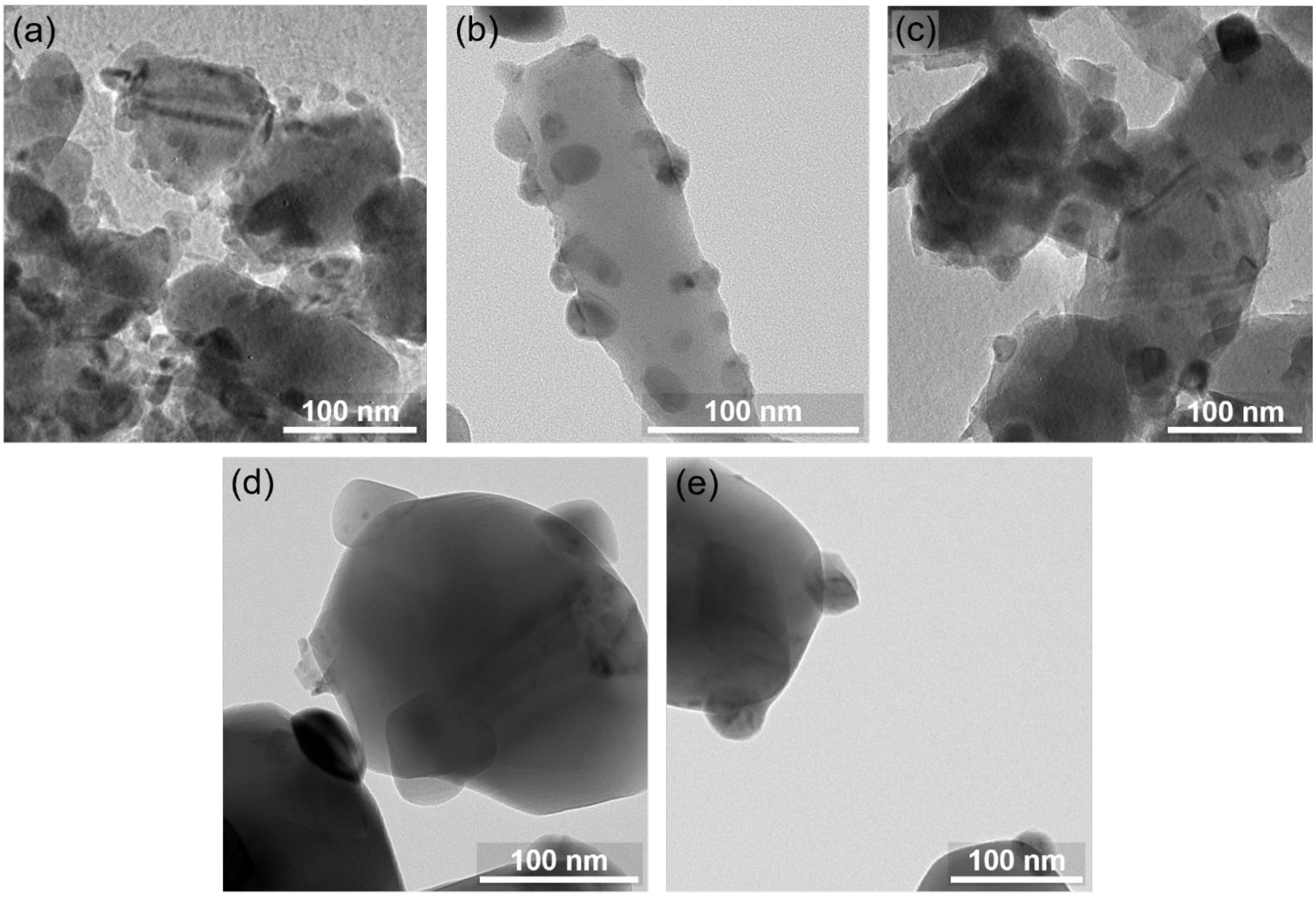

Figure S9: Representative TEM images of the as-prepared (a), reduced (b) and spent (c) Ni/ZnO catalysts, and of the calcined (d) and spent (e) NiO/ZnO catalysts.

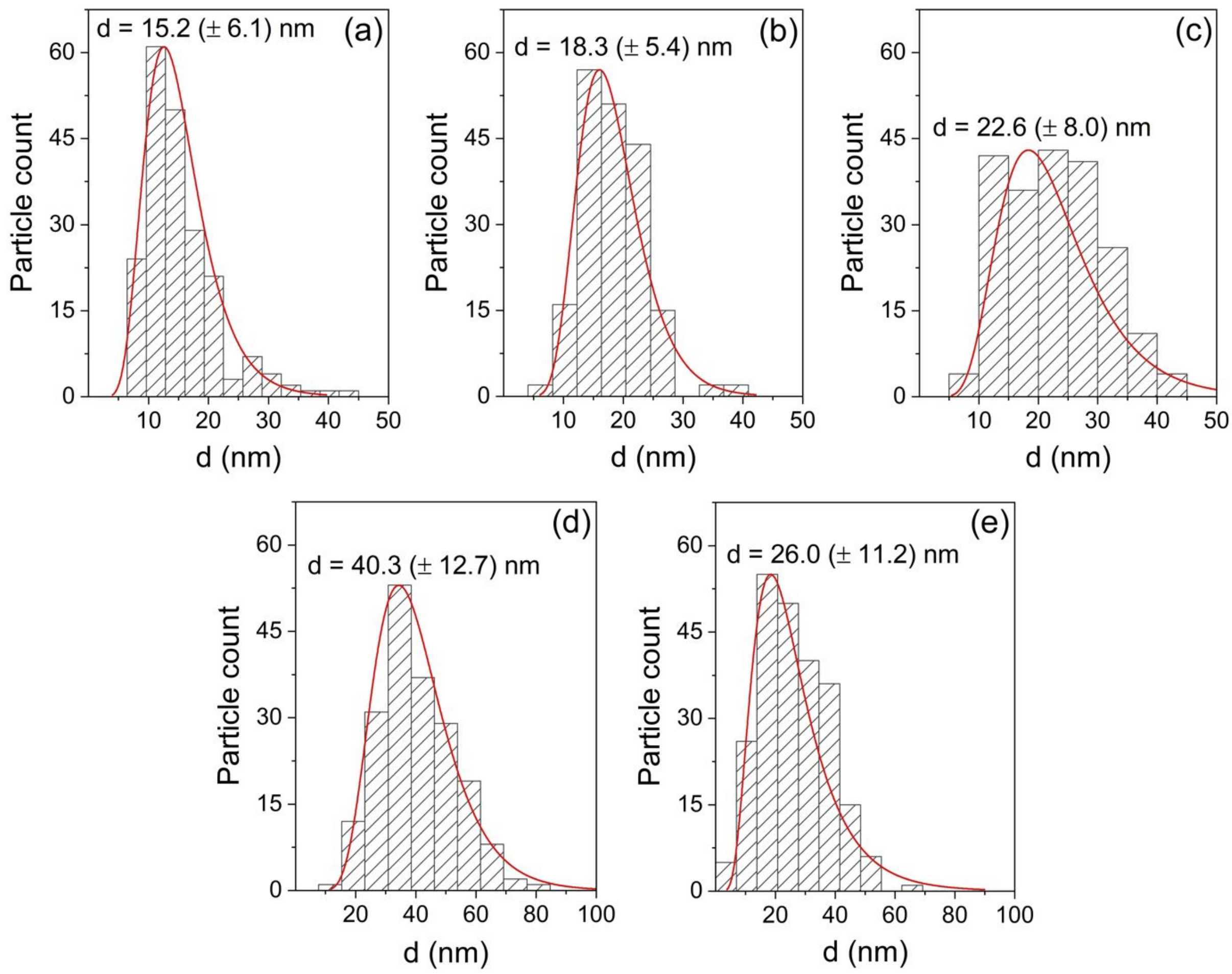

Figure S10: Histograms with log-normal probability density function (red line) of Ni-based particles size distribution for as-prepared (a), reduced (b) and spent (c) Ni/ZnO catalysts, and of the calcined (d) and spent (e) NiO/ZnO catalysts.

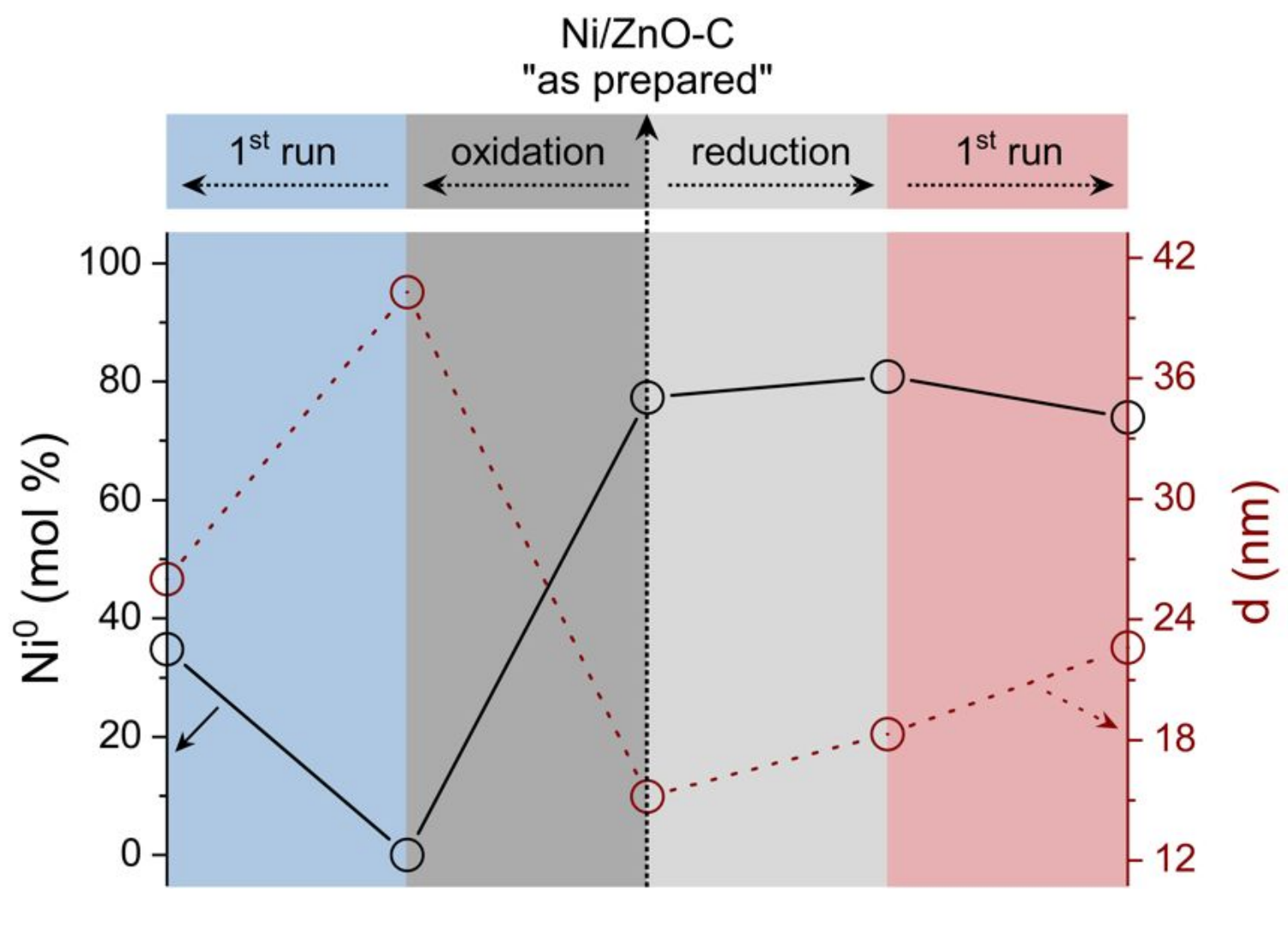

Figure S11: Comparison between the metallic Ni molar concentration estimated by Rietveld refinement and the Ni-based particles size estimated by particle counting in the TEM images.

Table S6: Elemental composition of the Ni catalysts in different life stages determined by ICP OES and elemental analysis.

Sample	Life stage	Atomic concentration (at. %)			Ratios		
		Ni	*Zn*	*C*	*C/Ni*	*C/Zn*	*Ni/Zn*
Ni/ZnO	*as prepared*	8.0	53.0	0.6	0.08	0.01	0.15
	after reaction	8.2	41.1	4.2	0.51	0.10	0.02
NiO/ZnO	*as prepared*	7.5	51.7	0.6	0.07	0.01	0.14
	after reaction	8.1	53.2	2.9	0.35	0.05	0.15

Table S7: Binding energies (BE) and atomic concentrations from the deconvolution of XPS spectra of the Ni/ZnO catalyst in different life stages. Calibrated to Zn $2p_{3/2}$ 1021.72 eV.

Peak	Deconvoluted peak	Life stage					
		as prepared		*Reduced*		*after reaction*	
		Bind. energy (eV)	*At. concn. (at. %)*	*Bind. energy (eV)*	*At. concn. (at. %)*	*Bind. energy (eV)*	*At. concn. (at. %)*
C 1s	*C–Ni*	—	—	283.3	9.5	283.2	47.4
	C–C	285.0	58.0	284.8	80.3	285.0	24.2
	C–O	286.6	11.2	286.2	1.0	286.5	4.6
	carbonates	289.1	30.1	288.7	9.4	289.3	23.8
O 1s	*O–Zn*	530.4	43.6	530.3	42.4	530.4	52.9
	OH-Zn	531.8	16.2	531.7	13.5	531.7	14.3
	O-Ni	528.8	20.8	529.0	25.8	529.2	2.1
	OH-Ni	530.4	13.1	530.7	13.8	530.9	24.9
	adsorbed water	532.7	6.2	532.6	4.5	532.7	5.8
Ni $2p_{3/2}$	*Ni^0*	852.9	8.3	852.3	22.9	852.3	100.0
	Ni–O	853.5	19.7	853.6	24.1	—	—
	Ni–OH	855.2	72.0	855.2	53.0	—	—
Zn $2p_{3/2}$	*Zn^0*	1020.3	13.3	1020.3	25.0	1020.6	19.0
	Zn–O	1021.7	69.0	1021.7	62.4	1021.7	64.8
	Zn–OH	1022.7	17.65	1022.7	12.5	1022.7	16.2

2. Thermodynamic Evaluation of the Direct Carboreduction Pathway (CO Atmosphere)

To complement the thermodynamic investigations presented in the main text and provide a comprehensive guide for alternative experimental synthesis conditions, we evaluated a chemical environment consisting of a pure CO atmosphere. In this scenario, the carbon monoxide acts simultaneously as the carbon source and the sole reducing agent (Direct Carboreduction), where the oxygen from the precursor (oxides) is discarded as gas-phase CO_2 rather than H_2O. The corresponding balance of mass for both the metallic nickel and nickel oxide precursor routes is described by Equations S1 and S2, respectively:

$$3Ni + ZnO + 3CO \rightarrow Ni_3ZnC + 2CO_2 \tag{S1}$$

$$3NiO + ZnO + 6CO \rightarrow Ni_3ZnC + 5CO_2 \tag{S2}$$

The calculated reaction energies (ΔE) derived from first-principles ground-state optimizations for this atmosphere are summarized in Table S1.

Table S8. Calculated reaction energies (ΔE, in kJ/mo) for Ni_3ZnC formation under a pure CO atmosphere (Direct Carboreduction pathway yielding CO_2).

Precursor Route	Output Gas	ΔE (kJ/mol)
Metallic Ni	CO_2	-298
Ni Oxide (NiO)	CO_2	-966

Both pathways are highly exergonic under a pure CO atmosphere. Crucially, the co-reduction pathway starting from NiO precursors exhibits an enhanced exothermicity compared to the metallic nickel route, yielding a significant downward energy shift of -668.00 kJ/mol. From an

atomic perspective, this pronounced driving force is a direct consequence of the exceptional thermodynamic stability of the generated CO_2 molecules during the sacrificial reduction of the initial NiO oxide lattice ($3NiO + 3CO \rightarrow 3Ni + 3CO_2$). These findings underscore that the in-situ growth of the Ni_3ZnC phase from mixed oxide precursors remains the most thermodynamically favored energetic pathway across different realistic reactive gas environments.

3. *Relaxed CO adsorption structures at Ni_3CZn catalyst and charge density differences for all adsorption sites.*

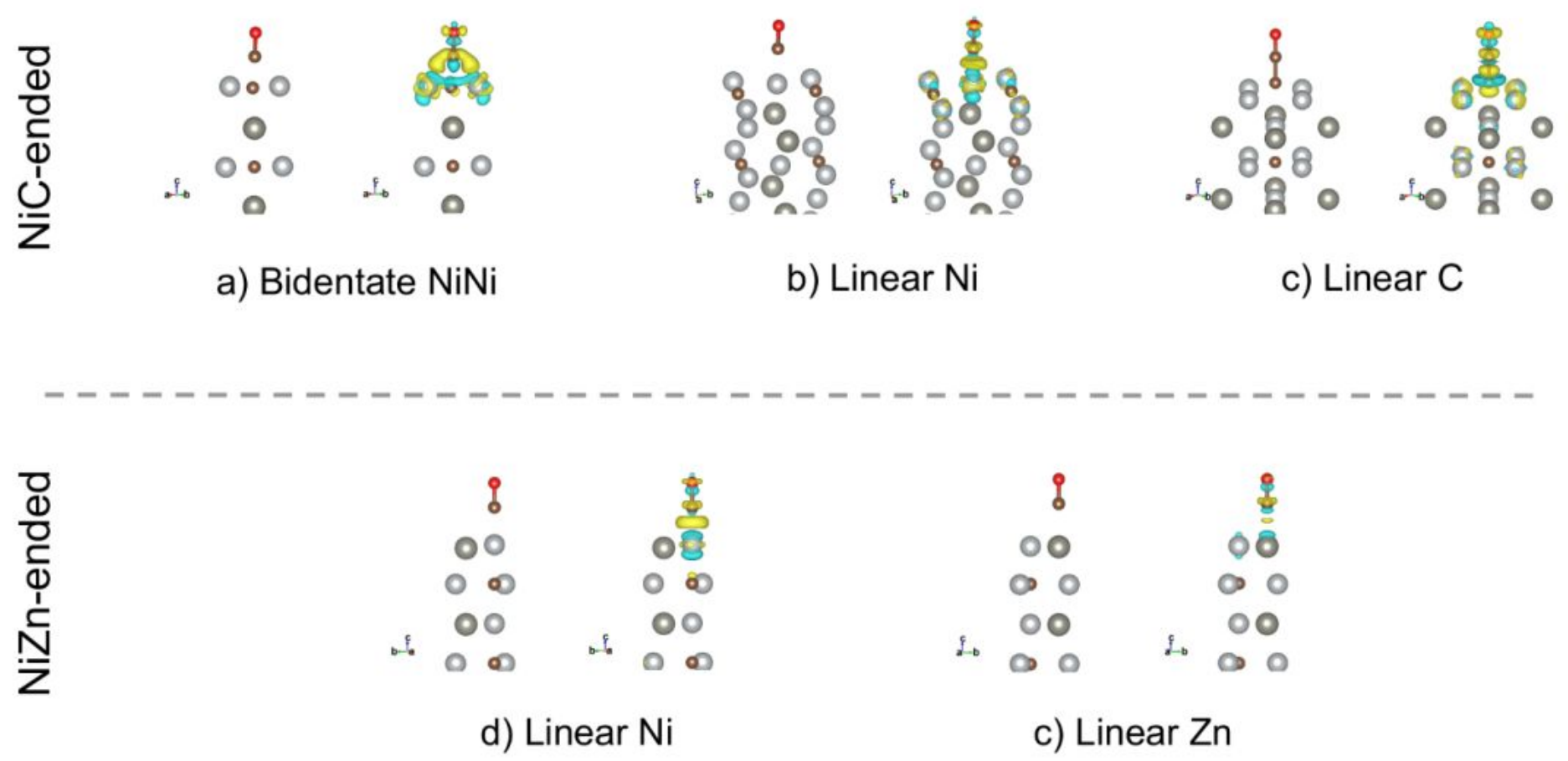

Figure S12: Relaxed CO adsorption structures at (100)-Ni_3ZnC surface and charge density differences. The nickel, zinc, carbon, and oxygen atoms are marked as light gray, gray, brown, and red, respectively. The blue and yellow isosurface represents charge accumulation and charge depletion regions. The isosurfaces are shown for 0.007 e/bohr^3.

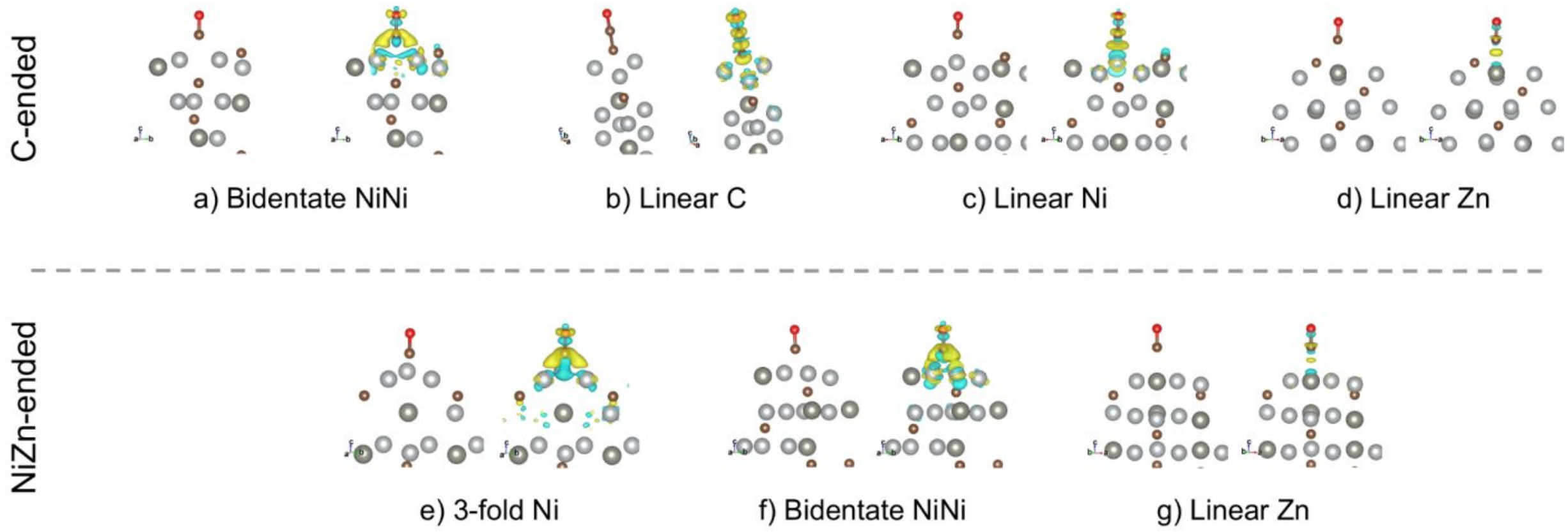

Figure S13: Relaxed CO adsorption structures at (111)-Ni_3ZnC surface and charge density differences. The nickel, zinc, carbon, and oxygen atoms are marked as light gray, gray, brown, and red, respectively. The blue and yellow isosurface represents charge accumulation and charge depletion regions. The isosurfaces are shown for 0.007 e/bohr^3.

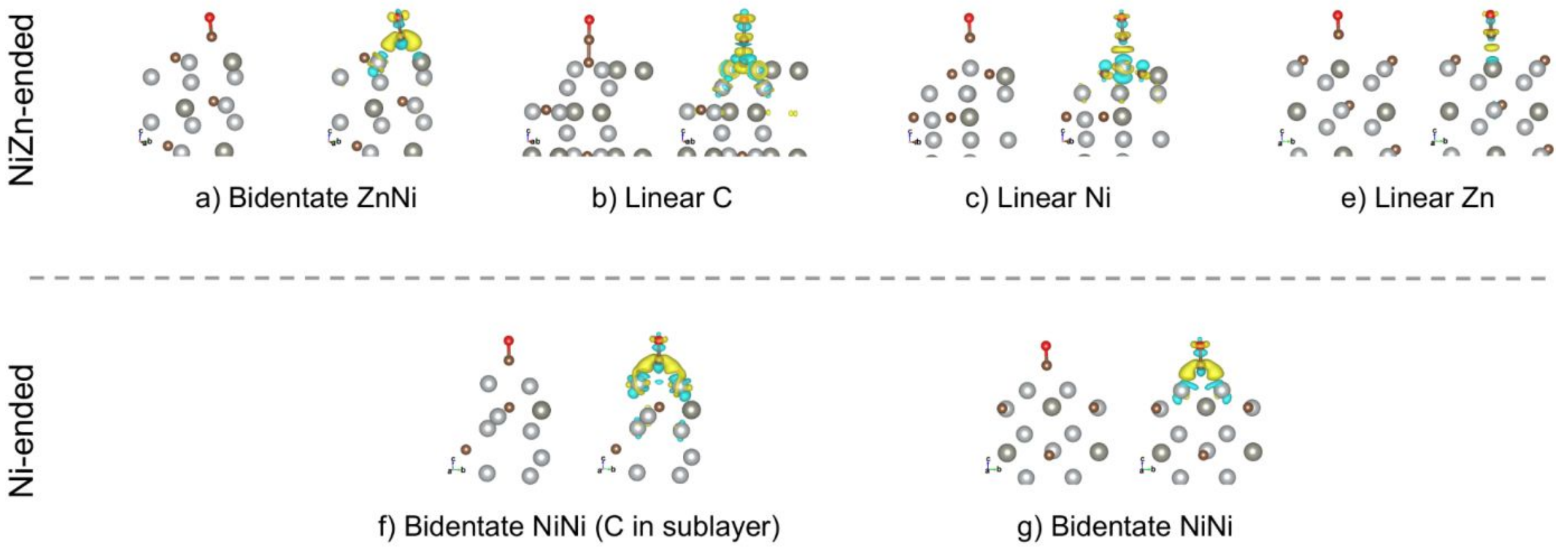

Figure S14: Relaxed CO adsorption structures at (110)-Ni_3ZnC surface and charge density differences. The nickel, zinc, carbon, and oxygen atoms are marked as light gray, gray, brown, and red, respectively. The blue and yellow isosurface represents charge accumulation and charge depletion regions. The isosurfaces are shown for 0.007 e/bohr^3.

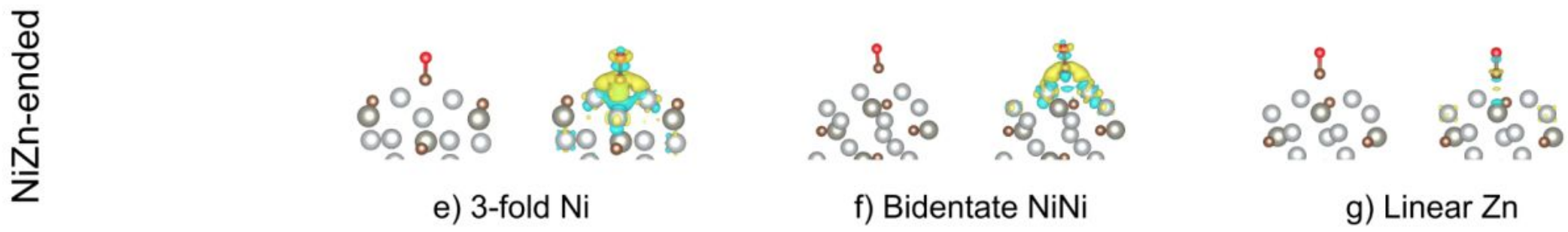

Figure S15: Relaxed CO adsorption structures at (211)-Ni_3ZnC surface and charge density differences. Due to the surface structure, the figure exhibits a stable termination. The nickel, zinc, carbon, and oxygen atoms are marked as light gray, gray, brown, and red, respectively. The blue and yellow isosurface represents charge accumulation and charge depletion regions. The isosurfaces are shown for 0.007 e/bohr^3.
